# Connectivity alterations in autism reflect functional idiosyncrasy

**DOI:** 10.1038/s42003-021-02572-6

**Published:** 2021-09-15

**Authors:** Oualid Benkarim, Casey Paquola, Bo-yong Park, Seok-Jun Hong, Jessica Royer, Reinder Vos de Wael, Sara Lariviere, Sofie Valk, Danilo Bzdok, Laurent Mottron, Boris C. Bernhardt

**Affiliations:** 1 McConnell Brain Imaging Centre, Montreal Neurological Institute and Hospital, McGill University, Montreal, QC Canada; 2 Center for the Developing Brain, Child Mind Institute, New York, NY USA; 3 Center for Neuroscience Imaging Research, Institute for Basic Science, Sungkyunkwan University, Suwon, South Korea; 4 Department of Biomedical Engineering, Sungkyunkwan University, Suwon, South Korea; 5 Max Planck Institute for Human Cognitive and Brain Sciences, Leipzig, Germany; 6 INM-7, FZ Jülich, Jülich, Germany; 7 Department of Biomedical Engineering, Faculty of Medicine, McGill University, Montreal, QC Canada; 8 Mila – Quebec Artificial Intelligence Institute, Montreal, QC Canada; 9 Centre de recherche du CIUSSSNIM et Département de Psychiatrie, Université de Montréal, Montreal, QC Canada

**Keywords:** Autism spectrum disorders, Computational neuroscience

## Abstract

Autism spectrum disorder (ASD) is commonly understood as an alteration of brain networks, yet case-control analyses against typically-developing controls (TD) have yielded inconsistent results. Here, we devised a novel approach to profile the inter-individual variability in functional network organization and tested whether such idiosyncrasy contributes to connectivity alterations in ASD. Studying a multi-centric dataset with 157 ASD and 172 TD, we obtained robust evidence for increased idiosyncrasy in ASD relative to TD in default mode, somatomotor and attention networks, but also reduced idiosyncrasy in lateral temporal cortices. Idiosyncrasy increased with age and significantly correlated with symptom severity in ASD. Furthermore, while patterns of functional idiosyncrasy were not correlated with ASD-related cortical thickness alterations, they co-localized with the expression patterns of ASD risk genes. Notably, we could demonstrate that patterns of atypical idiosyncrasy in ASD closely overlapped with connectivity alterations that are measurable with conventional case-control designs and may, thus, be a principal driver of inconsistency in the autism connectomics literature. These findings support important interactions between inter-individual heterogeneity in autism and functional signatures. Our findings provide novel biomarkers to study atypical brain development and may consolidate prior research findings on the variable nature of connectome level anomalies in autism.

## Abstract (web summary)

Benkarim et al devise an approach to profile inter-individual variability in functional network organization and test whether such idiosyncrasy contributes to the connectivity alterations found in Autism Spectrum Disorder. Their approach provides potential biomarkers to study atypical brain development and may be used to consolidate prior research findings on the variable nature of connectome level anomalies in autism.

## Introduction

Autism spectrum disorder (ASD) is one of the most common and persistent neurodevelopmental conditions. Behaviorally diagnosed on the basis of clinical observations and standardized tools assessing atypical communication, social interaction, and sometimes restricted and repetitive behaviors and interests^1^, the broad umbrella term of ASD has resulted in a steady increase in autism prevalence^2^. This increase in diagnostic sensitivity has on the other hand led to increasing recognition of the heterogeneity of diagnosed individuals^3–5^, and challenges for specificity^6^. This high variability is present at the phenotypic level of behavioral symptoms and at the level of genetic mechanisms previously associated with ASD^7–9^, and renders the study of autism particularly challenging. As etiology and pathophysiology remain largely unclear and similarly heterogeneous, efforts have increasingly shifted to neuroimaging techniques to identify intermediary autism phenotypes^10,11^. It is hoped that these can potentially consolidate molecular perturbations and behavioral perspectives on ASD and identify biomarkers of symptom severity.

Fueled by the increased availability of data sharing initiatives^12–15^, numerous neuroimaging studies based on resting-state functional magnetic resonance imaging (rs-fMRI) have indicated that autistic individuals often present with a mosaic pattern of connectivity alterations between distributed cortical regions relative to typically developing (TD) controls^12,16–19^. These connectivity alterations often manifest in the form of connectivity reductions in both higher order association cortices as well as sensory and motor regions, and sometimes co-occur with patches of connectivity increases between cortical and subcortical nodes^20^. However, other research has also emphasized (i) little overlap between reported results, (ii) variable patterns of hyper/hypo-connectivity, and (iii) an impact of preprocessing choices as well as subject-specific head motion and other confounds on observed findings^20–25^. Inconsistent findings have also been attributed to the use of conventional case-control designs in connectomics research in autism, which assume within-group homogeneity^26,27^. In addition to efforts that attempt to address this heterogeneity by subtyping ASD individuals into more homogeneous groups^4,5^, nascent literature has emphasized the importance to study interindividual variability of functional connectivity patterns in ASD compared to TD^28–30^. Interindividual variability in connectivity may logically follow the interindividual variability of activation previously demonstrated in perceptual and motor domains^31,32^. This body of work suggests that such idiosyncrasy may be an important feature of functional connectome organization in ASD, with greater variability in functional topography among ASD individuals relative to TD^32^. At the group level, this may potentially impact the analysis of connectivity differences between ASD and TD when assuming an identical alignment between the functional and structural domains among individuals. In other words, anatomical alignment does not guarantee correspondence of intrinsic functional profiles. Ignoring this phenomenon may lead to losing subject-specific features of network organization at the group level^33,34^. In ASD, given the highly idiosyncratic nature of the functional connectome, this is even more pronounced^29^, leading to spurious differences in connectivity that might be better explained when taking into consideration this heterogeneity^35,36^.

Although recent work has suggested an idiosyncratic organization of the functional connectome in ASD^28–30^, here we expand these approaches in several important ways. First, we developed a novel multi-marker profiling of idiosyncrasy, based on measures of spatial variability, connectome manifold analysis as well as probabilistic approaches to characterize the uncertainty of subject-specific functional topographies. These descriptors comprehensively profiled differences in idiosyncrasy between ASD and TD and provided the basis for an assessment of associations to age and symptom severity. To furthermore identify structural and potential molecular factors that give rise to the spatial patterns of ASD-related network idiosyncrasy, we correlated idiosyncrasy findings in ASD against MRI-based cortical thickness and curvature findings as well as postmortem gene expression information. Indeed, prior research has demonstrated atypical cortical development in ASD^37,38^, with genetic risk factors likely to play a major role in brain anatomy and connectivity abnormalities^39^. Finally, we tested our main hypothesis and assessed how idiosyncrasy may relate to connectivity alterations in ASD vis-a-vis healthy controls observed at the group level^40,41^. Specifically, we conducted a group-level analysis to study functional connectivity differences between ASD and TD, capitalizing on prior graph theoretical measures^22^, with and without considering idiosyncrasy.

## Results

**Fig. 1:**
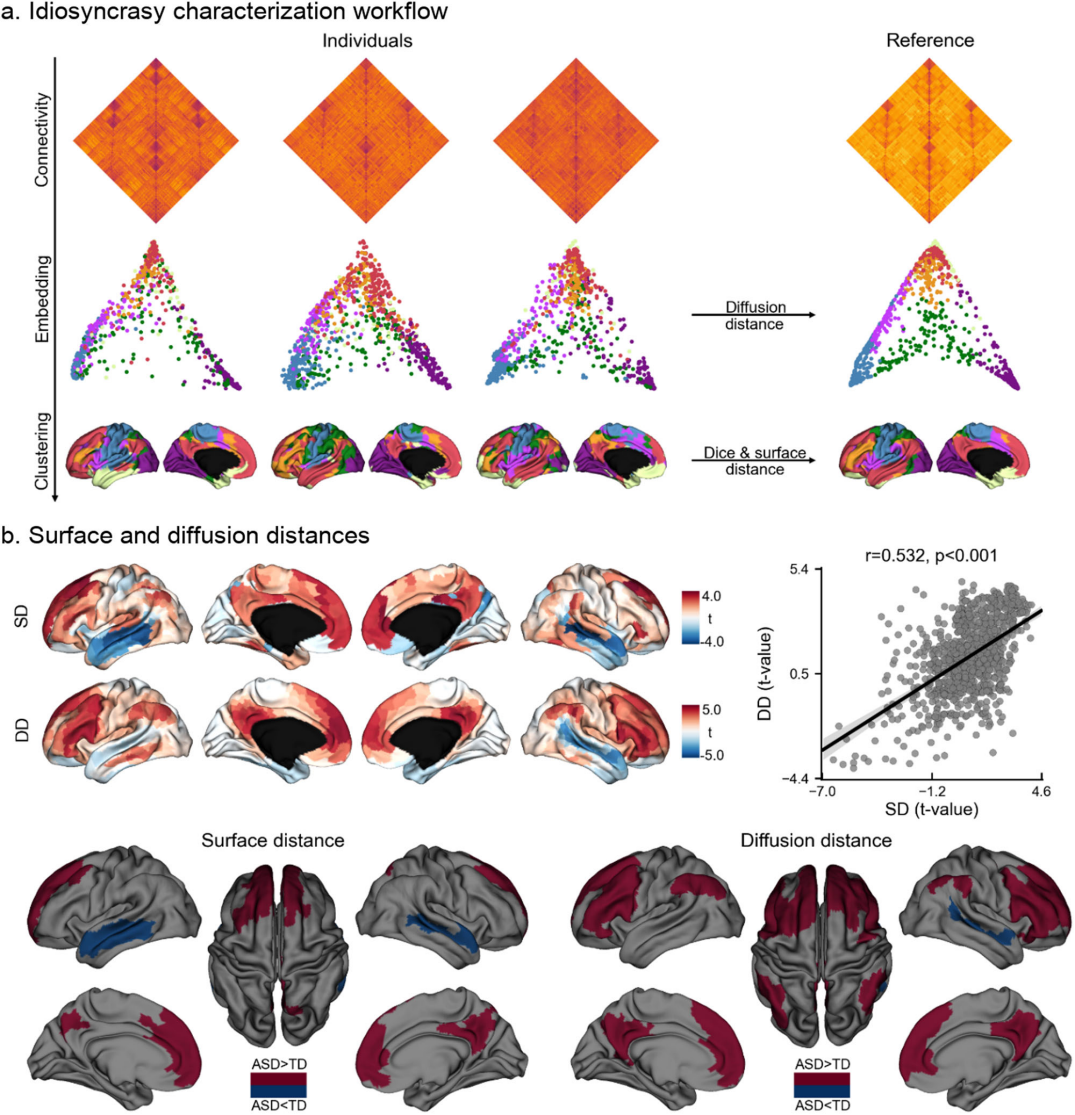
Spatial shifting and diffusion distance of intrinsic connectivity networks in ASD and TD. **a** Proposed workflow for intrinsic connectivity identification and idiosyncrasy characterization. **b** Statistical t-maps of surface (SD) and diffusion (DD) distance differences and their Pearson’s correlation (top), and areas showing significant idiosyncrasy differences in SD and DD (bottom). Shaded areas around the regression line denote a 95% confidence interval.

We studied idiosyncrasy based on rs-fMRI data from both waves of the Autism Brain Imaging Data Exchange (ABIDE I and II)^12,13^, a multisite data-sharing initiative. Specific site inclusion criteria and rigorous data quality control as in prior work^11,42,43^ resulted in a total of 329 participants (157/172 ASD/TD) from five different sites (see Supplementary Tables 1,  2). Our image processing strategy involved the mapping of functional signals to cortical surfaces as well as surface-based spherical alignment^44^, on which functional connectivity matrices were calculated at a single-subject level. Diffusion map embedding, a nonlinear dimensionality reduction technique that projects regions into a low-dimensional space governed by similarity in connectivity profiles^45,46^ identified a common low-dimensional manifold where individual embeddings were clustered into seven intrinsic connectivity networks (ICNs) using a Gaussian mixture model. Connectivity idiosyncrasy was characterized with two complementary features, namely the analysis of spatial shifting on the cortical surface meshes and the analysis of dispersion in connectome-based manifolds. Descriptors were computed relative to a reference embedding (and its corresponding clustering) built by averaging all individual connectivity matrices (see Fig. 1a). Findings corrected for the site, age, and sex unless otherwise specified. Further information about the dataset, image processing, and idiosyncrasy descriptors is provided in the Methods section.

### Idiosyncrasy is characterized by the shifting of functional networks in physical and embedding spaces

Idiosyncrasy was assessed through the quantification of surface distance (SD) and diffusion distance (DD). In brief, SD is the geodesic distance from a given point to the closest point in the corresponding reference network (see Fig. 1a). These geodesic distances were calculated along the cortical surface using Dijkstra’s algorithm^3,46^. DD, on the other hand, profiles idiosyncrasy in terms of the similarity in the connectivity patterns across individuals and with the canonical reference in the embedding space (see Idiosyncrasy descriptors section). Both SD and DDs are widely used measures that prior studies have shown to accurately capture differences in spatial and embedding domains^47–50^, respectively. Here, we leveraged these measures to provide a careful and complementary depiction of idiosyncrasy in terms of spatial shifting and connectivity similarity. Both approaches showed increased idiosyncrasy in ASD relative to TD in medial and lateral prefrontal regions in both hemispheres, with DD showing more marked effects bilaterally in the precuneus and angular gyrus (see Fig. 1b). Interestingly, ASD also showed bilateral reductions in idiosyncrasy compared to TD in lateral temporal cortices.

We complemented the surface-based analysis with an assessment of idiosyncrasy at the network level to determine if observed differences are related to spatial variability or to differences in the size of the ICNs. Here, we computed mean surface distance (MSD) to compare the locations of each ICN in each individual to its corresponding reference network (see Supplementary Fig. 1). Higher MSD values indicate that a given individual network deviates from the corresponding reference network. In both ASD and TD, the visual network (VN) showed the least idiosyncratic organization (i.e., lowest MSD), whereas the ventral attention network (VAN) had the most idiosyncratic organization (i.e., highest MSD). These results indicate that idiosyncrasy is network-specific. Comparing groups, we found significant differences after FDR correction in the dorsal attention (DAN, *p* = 0.005), default mode (DMN, *p* = 0.003), somatomotor (SMN, *p* = 0.009), and VAN (*p* = 0.002) networks, with ASD showing increased MSD relative to TD. Across the whole cortex, individuals with ASD also showed higher spatial shifting than TD (*p* = 0.002). Similar findings were also obtained when quantifying spatial shifting using Dice and Jaccard overlap measures. As higher MSD (and lower Dice/Jaccard) may also indicate that individual networks span larger/smaller portions of the cortex relative to the reference network because of hyper- or hypo-connectivity, we also analyzed between-group differences in network size. Notably, however, we did not find significant differences suggesting that findings were due to idiosyncrasy rather than connectivity differences per se (see Supplementary Table 3). Finally, all these measures are computed relative to a reference embedding that may have an impact on our results. In order to assess the robustness of our results to the reference embedding, we used bootstrapping to build different reference embeddings based on 50% of the samples in our dataset. Group-wise distributions of average Dice, Jaccard scores, and MSD across the whole cortex did not show any overlap (see Supplementary Fig. 2), indicating that our results are robust to the reference embedding.

**Fig. 2:**
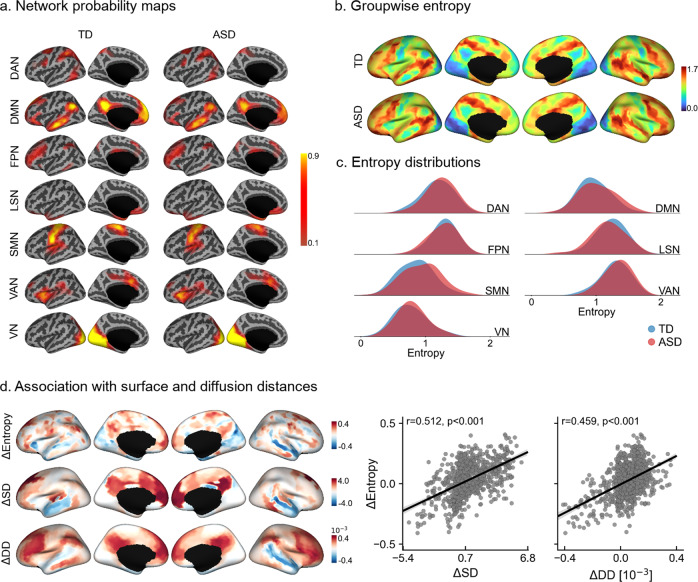
Spatial probability maps and entropy distributions of intrinsic connectivity networks. **a** Lateral and medial views of the left hemisphere displaying group-level spatial probability maps for each intrinsic connectivity network in TD (left) and ASD (right). **b** Group-wise entropy computed from the average probability maps for TD (top) and ASD (bottom). **c** Entropy distributions for each functional network (based on the reference clustering). **d** Spatial correlation of group-wise entropy differences (i.e., ΔEntropy) with the corresponding group-wise differences in surface (ΔSD) and diffusion (ΔDD) distances. Positive values reflect higher entropy/distance in ASD. Shaded areas around the regression lines denote a 95% confidence interval. DAN dorsal attention network, DMN default mode network, FPN frontoparietal network, LSN limbic system network, SMN somatomotor network, VAN ventral attention network, VN visual network.

We further contextualized idiosyncrasy using a probabilistic framework for each ICNs as shown in Fig. 2. Qualitatively, we observed more spreading of the spatial probability maps in ASD at the group-level, particularly in DMN and SMN (see Fig. 2a). As shown in Fig. 2b, this spreading is manifested as higher entropy in ASD (e.g., the same cortical location is assigned to different ICNs across individuals). Increases in ASD were highest in the SMN (Cohen’s *d* = 0.290), followed by VAN (*d* = 0.209), DAN (*d* = 0.203), and DMN (*d* = 0.188). On the other hand, the limbic system (LSN, *d* = −0.282) showed lower entropy in ASD (see Fig. 2c). We could observe high correlations of group-wise entropy differences with the corresponding differences in SD (*r* = 0.512, *p*_spin_ < 0.001) and DD (*r* = 0.459, *p*_spin_ < 0.001) (see Fig. 2d), even after accounting for spatial autocorrelation using nonparametric spin tests^51^. In accordance with the SD and DD findings, we observed lower entropy in ASD relative to TD in the lateral temporal lobe. Moreover, we also analyzed the potential link between idiosyncrasy and the hierarchical organization of the cortex. Specifically, we assessed the emergence of idiosyncrasy along the principal connectivity gradient^46^. As shown in Supplementary Fig. 3, idiosyncrasy increased following the principal gradient, from lowest in sensory/motor regions to highest in transmodal cortices.

Although the study site was included as a covariate in our main analyses, we repeated the SD and DD analyses for each site separately. Supplementary Figs. 4 and  5, respectively display global and site-specific SD and DD differences between TD and ASD. Despite variability in findings across sites, the overall direction of findings was relatively consistent across most of the included sites, particularly those with the highest numbers of subjects i.e., NYU, USM, and PITT (see Supplementary Table 2).

### Idiosyncrasy association to age and symptom severity

**Fig. 3:**
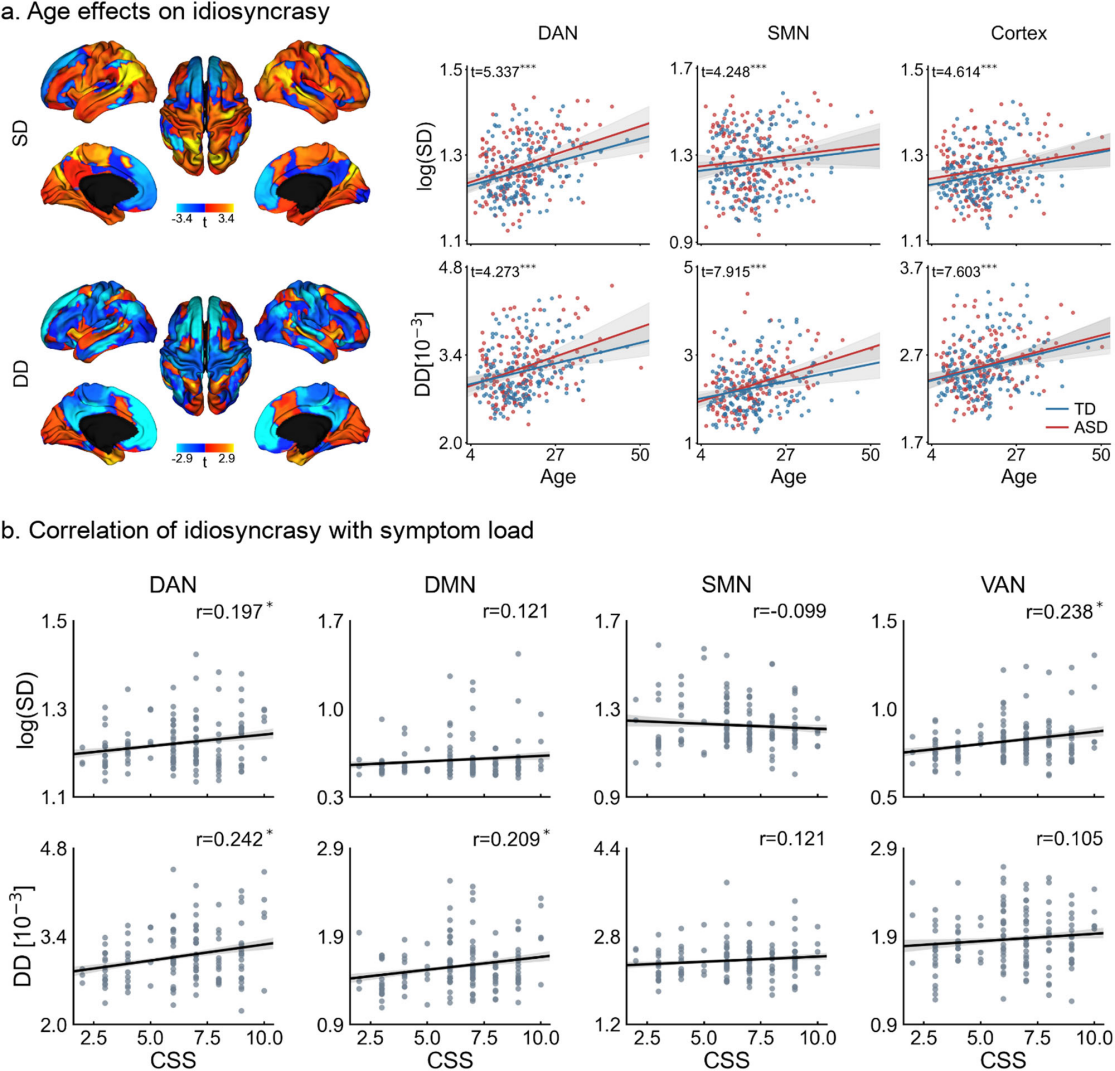
Idiosyncrasy association with age and symptom severity. **a** T-maps of age effects on surface and diffusion distances (left), and relationships of surface (top) and diffusion (bottom) distances with age for DAN and SMN, and globally for the entire cortex (right). **b** Pearson’s correlation of average surface (top) and diffusion (bottom) distances with calibrated severity scores (CSS) in networks with the highest idiosyncrasy (i.e., DAN, DMN, SMN, and VAN). Statistical significance is indicated with *, **, and ***, respectively denoting *p* < 0.05, *p* < 0.01, and *p* < 0.001 after FDR correction across seven networks for age and, for CSS, across the four different networks. Shaded areas around the regression lines denote a 95% confidence interval. DAN dorsal attention network, DMN default mode network, SMN somatomotor network, VAN ventral attention network.

When analyzing age effects (see Fig. 3 and Supplementary Fig. 6), we found significant associations to idiosyncrasy in the DAN (*p* < 0.001/0.001 for SD/DD), LSN (*p* = 0.089/0.001), SMN (*p* < 0.001 for both SD and DD), VAN (*p* = 0.016/0.001), and VN (*p* < 0.001). On the other hand, we found no significant relationship between DMN (*p* = 0.551/0.423) and FPN (*p* = 0.143/0.087). Overall, there was a significant effect of age on shifting in cortical and embedding spaces (*p* < 0.001), manifested in increasing SD and DD. These results indicate that idiosyncrasy increases with age. Nevertheless, ASD and TD showed similar slopes and there were no significant group-level interactions. We further assessed these results repeating our surface-based analysis using only the children (i.e., age <18 years) in our dataset and only the adults (i.e., age ≥18). Overall, results from these analyses, reported in Supplementary Fig. 7, were consistent with the findings obtained when using all individuals in our dataset. Nonetheless, when only using adults, the cluster in the temporal lobe showing higher idiosyncrasy in TD was relatively larger with respect to the cluster found in children.

We also investigated the association of our idiosyncrasy descriptors with ASD symptom severity based on the Autism Diagnostic Observation Schedule (ADOS). Specifically, we tested whether ADOS calibrated severity scores (CSS) were associated with SD and DDs (see Fig. 3b). The descriptors were computed for the networks that showed the highest idiosyncrasy and for the entire cortex. After correcting for multiple comparisons, significant associations were found in the default mode and attention networks, whereas SMN showed no significant association with CSS. Across the whole cortex, we found a significant association of CSS with DD (*r* = 0.208, *p* = 0.016) but not with SD (*r* = 0.113, *p* = 0.198). From these results, we can see that increasing idiosyncrasy is related to symptom severity.

### Associations to cortical morphology and gene expression patterns

**Fig. 4:**
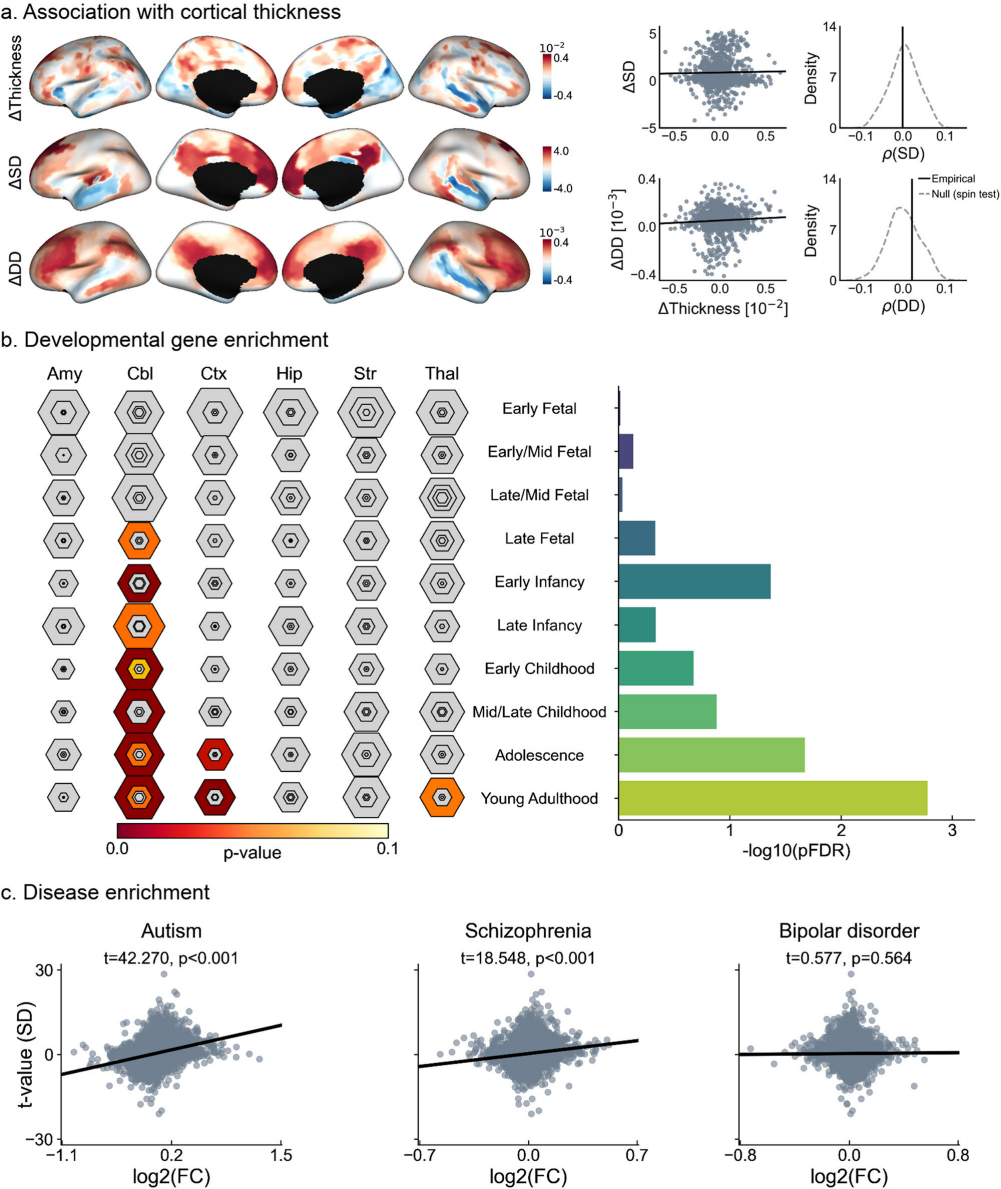
Associations to cortical morphology and gene expression patterns. **a** Correlation of group-wise differences in surface (top) and diffusion (bottom) distances with cortical thickness, and comparison of empirical correlation with the null distribution obtained using 1000 spin permutation tests to account for spatial autocorrelation. **b** Developmental cortical enrichment, showing enrichment mainly in the cerebellum, cortex, and striatum (left), specifically in adolescence and young adulthood (right). In the left panel, the size of hexagon rings represents the proportion of genes specifically expressed in a particular tissue at a particular developmental stage. Varying stringencies for enrichment with respect to specificity index threshold (pSI) are represented by the size of hexagons going from least (outer hexagon) to most specific (center hexagon) (pSI = 0.05, 0.01, 0.001, and 0.0001, respectively)^59^. Colors represent FDR-corrected *p* values. The right panel reports the log-transformed FDR-corrected *p* values, averaged across all brain structures. **c** Associations of gene expression in neuropsychiatric disorders, where log2(FC) stands for log2 fold-change of the genes in each disorder and the vertical axis indicates the significance of the relationship strength of the genes with idiosyncrasy in terms of surface distance (SD). Amy amygdala; Cbl cerebellum; Ctx cortex, Hip hippocampus, Str striatum, Thal thalamus.

Several studies^3,37,43,52–55^ have reported morphological alterations in ASD relative to TD. Cortical thickness changes were overall consistent with morphological anomalies reported in the literature, showing a mix of frontal and midline parietal cortical thickening sometimes together with patches of cortical thinning in temporal regions^37,43,52–54,56^. Importantly, we also inspected the relationship of the functional idiosyncrasy descriptors with changes in cortical morphology, by running spatial correlation analyses between group-wise differences in cortical thickness and mean curvature index to those in SD and DD. To account for spatial autocorrelations, we used spin tests with 1000 permutations^51^. As shown in Fig. 4a, we found no significant associations between cortical thickness and either measure of idiosyncrasy. Similar results were found when using a mean curvature index as a descriptor of cortical morphology, as shown in Supplementary Fig. 8. These results suggest that differences in functional idiosyncrasy are not spatially overlapping with potential alterations in cortical morphology in the ASD sample studied here.

Furthermore, we explored potential neurobiological correlates of idiosyncrasy in ASD. Idiosyncrasy maps obtained using SD and SD were correlated with postmortem gene expression maps from six donors provided by the Allen Institute for Brain Sciences (AIBS)^57^. Significant genes were identified by spatially correlating their expression patterns with our maps of idiosyncrasy based on spin tests with 1000 permutations, across the six postmortem cortical brain samples. Only genes that were significantly associated with idiosyncrasy and consistently expressed across all six donors (average inter-donor correlation ≥0.5) were considered for further analysis^58^. Selected genes (see Supplementary Table 4) were tested for developmental expression analysis across different developmental time windows^59^, from early fetal to young adulthood, and disease enrichment analysis. Developmental gene expression analysis highlighted associations of our idiosyncrasy descriptors with genes expressed in the brain from early infancy onwards (see Fig. 4b), and across several brain regions comprising the cerebellum, cortex, and striatum. Significant gene expressions were predominantly found in adolescence and young adulthood. Furthermore, we performed disease enrichment analysis to investigate the relationship between the strength of the association derived for each gene expression (with respect to our idiosyncrasy map) and a set of differential gene expression signatures in ASD, schizophrenia, and bipolar disorder. As shown in Fig. 4c and Supplementary Fig. 9, this analysis revealed that cortical patterns of idiosyncrasy were more strongly associated with differential gene expression^60^ in ASD (*t* = 42.270/28.099, *p* < 0.001 for SD/DD) than in schizophrenia (*t* = 18.548/14.192, *p* < 0.001) or bipolar disorder (*t* = 0.577/−2.014, *p* = 0.564/0.044).

### Connectivity alterations reflect idiosyncrasy

Prior research has suggested connectivity alterations in ASD relative to controls, but patterns of findings have overall not been consistent^28,29^. Here, we examined the spatial relationship between idiosyncrasy (in terms of SD and DD) and overall connectivity alterations, quantified using degree centrality (DC; see below for findings using eigenvector centrality). DC provides an unbiased depiction of the functional connectome that assigns each cortical location the number of connections exceeding a predefined threshold, set here to 0.2^22^.

**Fig. 5:**
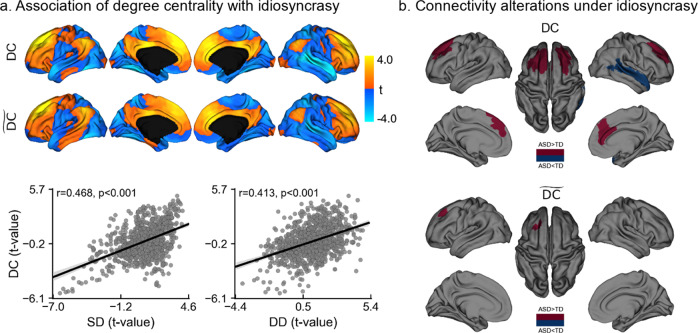
Association of degree centrality with idiosyncrasy. **a** Statistical t-maps (top) of areas showing differences in degree centrality between ASD and TD before (i.e., DC) and after controlling for idiosyncrasy (i.e., $\tilde{DC}$), and Pearson’s correlation (bottom) of DC t-map with t-maps of surface (SD) and diffusion (DD) distances. **b** Regions showing significant DC increases (red) and decreases (blue) in ASD before (top) and after (bottom) controlling for idiosyncrasy. Idiosyncrasy is represented with SD and DD as additional covariates. Shaded areas around the regression lines denote a 95% confidence interval.

The relationships of degree centrality with surface and diffusion distances are shown in Fig. 5a and reported in Table 1 for each ICN. DC showed strong correlations with both SD (*r* = 0.468, *p* < 0.001) and DD (*r* = 0.413, *p* < 0.001). For DC, positive/negative values indicate hyper/hypo-connectivity in ASD and higher/lower spatial deviations in surface and diffusion distances relative to TD. These results show that connectivity alterations in ASD are significantly associated with idiosyncrasy. Regions that exhibit hyper-connectivity (i.e., higher DC) in ASD show increased spatial deviation from the locations of the canonical networks. At the network level, SD was associated with DC in FPN, DMN, and VAN, whereas DD was associated with DC in all networks except the limbic system. Given the relationship of idiosyncrasy with DC, we set out to investigate the role of idiosyncrasy in the connectivity alterations observed in previous work. We first analyzed the differences in DC between ASD and TD and then repeated the same analysis controlling for idiosyncrasy. That is, we used both SD and DD as additional covariates in our analysis. As shown in Fig. 5b, the number of clusters showing significant differences is considerably reduced, with only one small region in the left frontal lobe remaining. Findings were replicated using a different centrality measure (i.e., eigenvector centrality^61^), as shown in Supplementary Fig. 10 and Supplementary Table 5. Eigenvector centrality assigns each node its corresponding entry in the eigenvector with the largest eigenvalue of the connectivity matrix. With eigenvector centrality, none of the regions showing significant differences in connectivity survived after controlling for idiosyncrasy. Altogether, these findings suggest that identifiable connectivity alterations in conventional ASD to control comparisons do, at least in part, emanate as a result of the high variability in the spatial locations of the ICNs.

**Table 1:** Relationship of idiosyncrasy, in terms of network-wise surface (SD) and diffusion (DD) distances, with average degree centrality for each intrinsic connectivity network.

	SD	DD
DAN	*t* = 1.57	*t* = 3.99^*^
DMN	*t* = 2.43^*^	*t* = 6.09^*^
FPN	*t* = 5.38^*^	*t* = 6.24^*^
LSN	*t* = −0.61	*t* = −1.10
SMN	*t* = −0.30	*t* = 4.35^*^
VAN	*t* = 3.94^*^	*t* = 5.15^*^
VN	*t* = −0.18	*t* = 3.13^*^

Significant associations after FDR correction are denoted with *.

## Discussion

Neurodevelopment is a complex yet coordinated process shaping the anatomy and function of multiple brain networks, with important variability across individuals. Characterizing this variability may add precision in the study of typical development and may advance our understanding of atypical neurodevelopment in diverse indications such as ASD^62,63^. Multiple studies have previously reported atypical functional connectivity in ASD, contributing to the overall notion of ASD as a disorder of brain networks^11,12,22^. However, there have also been reports questioning the consistency of findings, both in terms of which networks are involved and in terms of the directionality of findings^23,25,64^. Beyond an increasing recognition on the impact of preprocessing choices and sample inclusion criteria^4,6,25^, a growing research line is hinting at a more variable and idiosyncratic organization of the functional connectome in ASD as a potential contributor to these inconsistent findings^28–30^. In essence, idiosyncrasy describes an increased spatial variability in the mapping between functional network organization and brain anatomy. Here, we set out to (i) characterize such idiosyncratic network organization in ASD, using novel metrics that capture network variation in both physical and topological spaces, (ii) examine associations to age and symptom severity, (iii) explore morphological and genetic associations, and (iv) investigate how idiosyncrasy may contribute to functional connectivity alterations commonly seen in ASD to control case-comparison studies. In short, our findings suggest that ASD presents with a mosaic of idiosyncrasy alterations relative to TD, with mainly increases in ASD, together with focal decreases in network idiosyncrasy in lateral temporal regions. Idiosyncrasy was found to relate to both age and symptom load as measured with ADOS CSS^65–67^, and the spatial topography of ASD-related network idiosyncrasy strongly correlated with the expression of autism risk genes. Notably, we could also show that idiosyncrasy contributes to ASD versus TD connectivity differences that are detectable with typical case-control analysis, motivating future research strategies that consider patterns of idiosyncrasy in their analyses.

Core to our work were two complementary approaches to quantify functional idiosyncrasy, with one approach operating in the spatial domain and another one in connectivity-determined manifold spaces. Both approaches converged in showing that while functional network organization is idiosyncratic in both TD and ASD, the latter showed a mosaic of mainly increases in network idiosyncrasy across multiple functional systems together with patches of idiosyncrasy reductions. In the spatial domain, we compared individual network locations to a canonical reference connectome, built by averaging all individual connectivity matrices in our dataset. This highlighted that several networks (i.e., DAN, DMN, SMN, and VAN) were shifted in ASD from the typical locations of their corresponding reference networks. Complementing idiosyncrasy profiling in the spatial domain, we characterized idiosyncrasy in a connectivity-informed manifold space. Such manifolds provide coordinate systems based on intrinsic network organization and are, thus, decoupled from the underlying anatomy^46,68,69^. In several recent studies, our group and others capitalized on manifold spaces to represent structural and functional connectome information^70–72^, to assess structure-function coupling^72^, and to study typical and atypical connectome organization^11,42,71,73,74^. Unlike their spatial counterparts, manifold-based idiosyncrasy measures tap into intersubject correlations^75^, and in turn, provide a metric sensitive to regional connectivity, as well as similarity in connectivity to other regions^76^. This descriptor converged with our SD analysis, in that it pointed to a spatially varying pattern of idiosyncrasy, with ASD showing mainly increased idiosyncrasy across multiple networks, encompassing sensory as well as higher-order networks. In addition to the convergence in findings across these two descriptors, we could cross-validate our findings using an entropy-based descriptor at the network level. This approach provided an independent probabilistic context to understand our findings in terms of interindividual spatial network uncertainty (i.e., networks with high interindividual variability show high entropy), supporting increased idiosyncrasy in ASD in multiple networks relative to TD. Note that these descriptors were used to study idiosyncrasy at the cortical level. Besides cortical regions, however, subcortical areas and subcortico–cortical interactions play an important role in ASD^20,42^. The incorporation of subcortical regions may have important implications for our idiosyncrasy descriptors, and it may further enrich the description of network hypo/hyper-connectivity observed in group-level contrast analysis. With the exception of distance-based measures (i.e., SD and MSD), our descriptors could be easily extended to incorporate and account for differences in subcortical connectivity patterns.

The functional networks found to be idiosyncratic in our analyses have been consistently shown to diverge in analyses that compared functional connectivity in ASD relative to TD at the group level. Indeed, several studies have reported connectivity alterations in ASD individuals relative to TD in the DAN^77–79^, VAN^78,80^, DMN^5,77,81,82^, and the SMN^12,20,22,82^. Our findings show that the degree of spatial shifting, irrespective of the cohort (i.e., in both ASD and TD), is distributed across the putative functional hierarchy, affecting primary sensory, unimodal association, and attentional, as well as higher-order transmodal systems such as the DMN. Interestingly, and previously unreported in ASD, our two idiosyncrasy measures also pointed to reduced idiosyncrasy in a region encompassing the lateral temporal lobe in ASD. A prior rs-fMRI study in neurotypical individuals^83^ found the lateral temporal cortex to be among the areas with the highest intersubject variability in intrinsic functional connectivity. Moreover, lateral temporal cortical areas have previously been suggested to show abnormal structural connectivity in ASD in very young children at risk for ASD^84^. In that study, lower structural network efficiency of primary and secondary auditory cortices was related to autism risk in children as young as 6 months old, and network inefficiencies were related to symptom load at a later follow-up^84^. The authors suggested that atypical organization in sensory systems in autism may manifest early, and potentially cascade into the organization of higher order networks—a finding in line with the sensory first hypothesis of autism and other neurodevelopmental disorders^85–87^. Although our findings are overall indicative of a relatively broad functional perturbation affecting many networks, using the principal connectivity gradient as a model of human cortical hierarchical organization, we were able to demonstrate an overall higher increase in idiosyncrasy in transmodal networks compared to sensory/motor networks, showing that increased idiosyncrasy in ASD is preferentially located in cortical regions with the most variability among neurotypical individuals, with the exception of the lateral temporal areas^83^. An increasing body of neuroimaging work has shown that primate and human cortical microstructure and function generally follows sensory-transmodal hierarchies^46,71,88,89^, recapitulating earlier models of primate cortical organization^90,91^. Further evidence on a hierarchical organization is supplied by electrographic and neuroimaging studies, showing similar gradients of temporal hierarchies in the primate cortex that follow sensory-transmodal hierarchies^88,92^. Local alterations at specific nodes along these hierarchies could ultimately affect integrative and heteromodal networks, such as the DMN, disproportionately and manifest as increased idiosyncrasy in these networks.

Findings of increased idiosyncratic organization in ASD are consistent with prior work reporting higher inconsistency in the incorporation of individual anatomical locations to the DMN and SMN^30^, and increased spatial shifting in DAN and VAN^28^. In our study, these four ICNs had a more idiosyncratic functional organization in ASD. Moreover, and similar to prior work, we found that idiosyncrasy increased with symptomatology, more specifically in social and communication difficulties, indicating that functional network reorganizations which diverge most from the normative group are reflected in more pronounced patterns of behavioral divergence on standardized testing. Nonetheless, these prior studies have largely overlooked the relationship of the underlying spatial topography to connectivity differences in ASD versus control populations. In^29^, it was shown that the existence of topographical distortions among individuals leads to a regression to the mean effect at the group level. In other words, the study of functional connectivity at the group level may be affected by latent misalignments between the functional organization and the underlying anatomy, potentially giving rise to spurious differences. Indeed, our analysis of functional connectivity alterations showed that idiosyncrasy is a potential confounder. Hyper- and hypo-connected regions found in ASD using degree and eigenvector centrality measures show great overlap with previous findings in multiple large-scale datasets using degree centrality^22^. However, after controlling for idiosyncrasy (using both surface and diffusion distances as covariates), differences were considerably reduced. A small patch with increased connectivity survived when using degree centrality, whereas with eigenvector centrality no connectivity differences were found. It is plausible that idiosyncratic reorganization in ASD breaks down the functional correspondence between homologous anatomical regions across individuals assumed in case-control studies, and thus challenges inference as well as the interpretation of previously reported connectivity differences. Intersubject variability in functional connectivity has been shown to be related to the variability in the position of functional regions even in normative populations^93^. This is closely related to an emerging literature on precision neuroimaging in healthy populations, where several studies have also shown specific within-subject features of network organization that do not manifest at the group level due to this effect^33,34,94,95^. In addition to potential spatial uncertainty, other findings have also shown that some of the connectivity alterations found in ASD are partially driven by short-term temporal variability^96^. Taken together, our findings suggest a marked influence of network idiosyncrasy on what is detectable with traditional case-control connectivity analyses. As such, they support the development of novel approaches to analyze connectivity differences at the group level, while also considering subject-specific variability, especially in atypical populations such as ASD.

Cortex-wide correlation analyses revealed no significant associations between differences in cortical morphology (quantified via cortical thickness and mean curvature index) and our maps of idiosyncrasy, ruling out a systematic relationship between alterations in both brain structure and function. We note, however, that in our sample, the lateral temporal lobe showed subtle degrees of cortical thinning in ASD compared to TD, which is in line with prior studies^55^ and spatially coincides with our findings of reduced idiosyncrasy in the lateral temporal areas in ASD. Albeit speculative, it is possible that cortical thinning in ASD in these regions may ultimately have downstream effects on functional connectivity (e.g., if the thinning relates to synaptic alterations and or subtle disconnection in the temporal lobe), and may thus relate to region-specific alterations in functional idiosyncrasy. The relationship of idiosyncrasy with morphology may be further investigated in future work using other MRI-derived measures, notably those sensitive to myeloarchitecture and tissue microstructure, which can be used in the study of structure-function association based on depth specific variations in cortical microstructure and to track developmental change^71,97^. On the other hand, correlating idiosyncrasy measures with age indicated an age-related increase in both ASD and TD, with no significant differences in trajectories between groups. As such, our results point to an increased functional network idiosyncrasy in ASD relative to controls already present at an early age, with neither a considerable aggravation nor normalization throughout childhood development, adolescence, and early adulthood. Notably, while our inclusion criteria allowed the study of both children and adults with ASD and TD, our youngest participants were 5 years old. In light of emerging studies suggesting connectivity anomalies in very young children with autism^84^, it will therefore be of relevance to assess network idiosyncrasy in small kids and infants and to also model intraindividual trajectories longitudinally. This will offer a more precise understanding of early mechanisms contributing to idiosyncrasy, alongside a more direct mapping of intraindividual trajectories in idiosyncratic networks.

Although our findings showing a mosaic pattern of increased and decreased idiosyncrasy warrant further investigation, a plausible explanation for this large-scale functional reorganization in ASD together with increased variability in both spatial and connectome-based network embeddings may relate to compensatory plasticity mechanisms and its imbalance in autism. By integrating genetic, cognitive, and neuroimaging findings, the so-called trigger threshold target model of autism^98^ has postulated that ASD may relate to neurodevelopmental disturbances that trigger compensatory reallocations of neural resources in autism. As a result, intact regions assume functions from nearby impaired areas. To accommodate this shifting of competences, spatially adjacent networks might be required to adjust their locations and/or typical functional crosstalk, a phenomenon that may contribute to the observed increases in SD and DD in the cohort with autism. Since reallocations are likely to occur within the same hemisphere (e.g., involving spatially adjacent networks), this shifting may give rise to increased distortions in homotopic interhemispheric connectivity because it breaks the functional interhemispheric correspondence, which is in line with previous findings^29^. Supporting this shifting of competences, prior work has suggested abnormal cortical plasticity in ASD^99^, with multiple genetic factors involved in this process. In fact, most genetic risk factors associated with ASD appear to be implicated in synaptic plasticity and connectivity more generally^100,101^. Genetic influences on functional connectivity are well established in both adults, and TD children and adolescents^102–104^. Prior imaging-genetics studies have consistently demonstrated considerable heritability of resting-state functional networks^103,105–107^. Moreover, these risk factors may be shared across a range of neuropsychiatric disorders^108^. The vast genetic diversity associated with ASD in conjunction with its high heritability^100,109^ may therefore account for heterogeneity in connectivity alterations observed in ASD^110^. In this work, we investigated the relationship of idiosyncrasy with gene expression, showing that genes associated with idiosyncrasy differences were more strongly correlated with differential gene expression in ASD than in schizophrenia and bipolar disorder, which further highlights idiosyncrasy as an important feature of autism. Note, however, that gene expression used in this analysis is derived from adult postmortem data from a different dataset (i.e., Allen Human Brain Atlas [AHBA]), and our findings may thus only represent indirect associations that need to be confirmed as additional resources and datasets become available that offer both neuroimaging and gene expression data in the same ASD and control populations. Besides genetic factors, the environment also plays an important role in shaping functional network organization throughout development^103,111^. Equally, environmental factors have been suggested to be a risk of neurodevelopmental disorders such as ASD^112–114^, and environmental factors may also contribute to the observed functional network idiosyncrasy in ASD. Of note, idiosyncratic network organization could be identified in the absence of any goal-oriented task in the current study, purely based on a task-free functional imaging acquisition. Although changes in these patterns might occur under different task conditions or mental states, this idiosyncrasy can be seen as an inherent characteristic of ASD brain organization that may contribute to unconstrained cognitive processes, during routine behavior, as well as specific tasks^29^. Moreover, the emergence of idiosyncrasy may be related to the way ASD individuals interact with external environments. Altered interactions with the environment may account for individual differences. For example, given cognitive inflexibility that has been reported in ASD^115^, idiosyncratic functional reorganizations may stem from compensatory mechanisms developed to overcome cognitive and behavioral rigidity^116^.

To conclude, our work characterized functional idiosyncrasy in spatial and connectivity-informed manifold dimensions of the functional connectome. Studying a large dataset of TD and ASD, our novel descriptors reliably captured differences in both groups, suggesting a mosaic pattern of idiosyncrasy increases and decreases in several functional networks in ASD. In addition to showing associations to age, symptom severity, as well as gene expression patterns, our findings notably indicated a marked relationship between idiosyncrasy and connectivity differences that can be identified using case-control analysis, which may consolidate some of the heterogeneity observed in previous studies in ASD and calls for the consideration of idiosyncrasy when studying the functional connectome in autism, since connectivity alterations may, at least partly, reflect an underlying idiosyncratic organization.

## Methods

### Participants and data acquisition

We studied rs-fMRI data from both waves of the openly-shared Autism Brain Imaging Data Exchange initiative (ABIDE I and II; http://fcon_1000.projects.nitrc.org/indi/abide)^12,13^. For our study, we selected those sites with ≥10 individuals per group and with both children and adults. After detailed quality control, only cases with acceptable T1-weighted (T1w) MRI, surface-extraction, and head motion in rs-fMRI were included in our analyses, resulting in a total of 329 subjects (157/172 ASD/TD, with mean ± SD age in years = 18.4 ± 8.2/18.4 ± 7.7) from five different sites: (1) NYU Langone Medical Center (NYU, 35/51 ASD/TD from ABIDE-I, and 21/19 from ABIDE-II); (2) University of Utah, School of Medicine (USM, 49/37 ASD/TD); (3) University of Pittsburgh, School of Medicine (PITT, 19/20 ASD/TD); (4) Trinity Centre for Health Sciences, Trinity College Dublin (TCD, 12/16 ASD/TD); and (5) Institut Pasteur/Robert Debré Hospital (IP, 11/21 ASD/TD). High-resolution T1w images and rs-fMRI were acquired on 3 T scanners from Siemens (NYU, USM, PITT) or Philips (IP, TCD). More information about acquisition settings for each site is provided in Supplementary Table 1.

Individuals with ASD were diagnosed by an in-person interview with clinical experts and gold standard diagnostics of the ADOS^117^ and/or Autism Diagnostic Interview-Revised (ADI-R)^118^. TD individuals did not have any history of mental disorders. For all groups, participants who had genetic disorders associated with autism (i.e., Fragile X), contraindications to MRI scanning, and pregnancy were excluded. The ABIDE data collections were performed in accordance with local Institutional Review Board guidelines, and data were fully anonymized. Written informed consent was obtained from all the participants. Detailed demographic information from participants included in our study are reported in Supplementary Table 2.

### Data preprocessing

T1w MRI data were preprocessed with FreeSurfer v5.1^44,119,120^. The pipeline performed automated bias field correction, registration to stereotaxic space, intensity normalization, skull-stripping, and tissue segmentation. White and pial surfaces were reconstructed using triangular surface tessellation and topology-corrected. Surfaces were inflated and spherically registered to *fsaverage*. For the rs-fMRI, we used preprocessed data previously made available by the Preprocessed Connectomes initiative (http://preprocessed-connectomes-project.org/abide). The preprocessing was performed with C-PAC (https://fcp-indi.github.io) and included slice-time correction, head motion correction, skull stripping, and intensity normalization. The rs-fMRI data were de-trended and nuisance effects related to head motion, white matter, and cerebrospinal fluid signals were removed using CompCor^121^, followed by band-pass filtering (0.01–0.1 Hz). Finally, rs-fMRI and T1w data were coregistered in MNI152 space using linear and nonlinear transformations. Individual rs-fMRI data were mapped to the corresponding mid-thickness surfaces, resampled to the Conte69 template (https://github.com/Washington-University/Pipelines), and smoothed using a 5 mm full width at half maximum (FWHM) kernel. All segmentations and surfaces were visually inspected. Subjects with erroneous segmentations or framewise displacements greater than 0.3 mm were excluded from our analyses.

### Identification of intrinsic connectivity networks

To identify and quantify idiosyncrasy in functional network organization, we mapped the rs-fMRI data to a low-dimensional space using the following steps (see Fig. 1a). First, we built the connectivity matrices from the rs-fMRI time-series of each individual in our dataset using linear correlation coefficients. The connectivity matrices were based on a functional parcellation with 1000 labels^50^, Fisher’s z-transformed and thresholded to only keep the 10% of the most similar entries per row^122^. We used diffusion mapping introduced in ref. ^45^, as implemented in BrainSpace^122^, to embed the rs-fMRI data into a low-dimensional manifold. This approach is robust to noise and computationally efficient compared to other nonlinear manifold learning techniques^123,124^. Briefly, diffusion mapping embeds the data into a particular Euclidean space in which the usual Euclidean distance corresponds to the diffusion distance on the data at a given scale or diffusion time. In this new space, interconnected cortical regions are nonlinearly projected to fall close to each other, whereas weakly connected regions are mapped to distant locations in the eigenspace. For our study, the diffusion time was set to 1, and the α parameter, which controls the influence of the density of sampling points on the manifold (from maximal influence α = 0, to no influence at all α = 1) was set to α = 0.5 to retain the global relations between data points in the embedded space, following prior work^11,46,125^. Since diffusion maps capture the main structures of the data along a few cardinal dimensions, we selected the first 30 eigenvectors similar to a previous study, corresponding to the largest eigenvalues to represent each individual embedding^126^.

To assess differences between TD and ASD, we averaged the connectivity matrices of all the individuals in our dataset to build a mean connectivity matrix, which was subsequently used to construct a reference embedding. This reference embedding was used as a representation of the canonical functional connectivity template. Because diffusion mapping may take the individual datasets into different Euclidean spaces, the standard Euclidean distance between the elements of these spaces is not meaningful. To bring the data into the same Euclidean space, we used a change of basis operator to map all the individual embeddings to the reference embedding^127^. In this way, we can compute the Euclidean distance within and between datasets, allowing us, therefore, to compare the individual diffusion maps to the reference embedding.

Finally, to identify the ICNs, all embeddings (including the reference) were clustered into seven components using a Gaussian mixture model with a full covariance matrix. Each point in the embedding was assigned to the cluster corresponding to the highest a posteriori probability. The mixture model was initialized with the seven ICNs proposed in^128^.

### Idiosyncrasy descriptors

Two different approaches were proposed to characterize idiosyncrasy, namely: spatial- and manifold-based distance measures. For the spatial measure, we used SD, which was computed for each point as the geodesic distance to the closest point in the corresponding reference network^3,46^. The second measure to characterize idiosyncrasy is based on diffusion distance, which is approximated using the Euclidean distance in the eigenspace between points of each individual to the reference embedding, such that points that fall far apart from their corresponding reference points show a high difference in their original rs-fMRI time-series. Instead of computing the distance between pairs of points, however, we take advantage of the clustering and compute the diffusion distance from a given point to its closest point in the reference embedding that belongs to the same cluster (i.e., ICN). Let ${\Psi }_{{{{{{\rm{c}}}}}}}^{{{{{{\rm{r}}}}}}}$ be the set of points of the reference embedding in cluster *c*, for each point ${\phi }_{c}\in {\varPhi }_{{{{{{\rm{c}}}}}}}^{{{{{{\rm{i}}}}}}}$ of the individual embedding *i* in the same cluster, our diffusion-based idiosyncrasy descriptor is then computed as follows:

1 $$dd({\phi }_{c}{,{{{{{\rm{\psi }}}}}}}_{{{{{{\rm{c}}}}}}}^{{{{{{\rm{r}}}}}}})=\Vert {\phi }_{c}-{{\psi }_{c}}\Vert^{2}.$$

In this way, this descriptor captures the variability that exists in the connectivity patterns that characterize a specific ICN among all the individuals in our dataset. Furthermore, to assess that idiosyncrasy differences are not related to the size of the ICNs, which would rather indicate alterations in connectivity, we aimed to quantify the spatial variability at the network level by computing the overlap and the extent of shifting of each individual clustering from the canonical reference for each ICN. To do so, we used the Dice similarity coefficient^129^, which is in common use in neuroimaging research:^130^

2 $${{{{{\mathrm{Dice}}}}}}\,(A,B)=\frac{2|A\cap B|}{|A|+|B|},$$

Jaccard index:

3 $${{{{{\mathrm{Jaccard}}}}}}\,(A,B)=\frac{|A{\cap }^{}B|}{|A{\cup }^{}B|},$$

and MSD:

4 $${{{{{\mathrm{MSD}}}}}}\,(A,B)=\frac{1}{|A|+|B|}\left(\mathop{\sum }\limits_{a\in A}d(a,B)\mathop{\sum}\limits_{b\in B}d(b,A)\right),$$

where *A* and *B* are respectively the reference and individual clusters corresponding to a specific network, |·| denotes cardinality, and *d*(*a*,*B*) is the geodesic distance between point *a* in cluster *A* to the closest point in cluster *B*. In this case, the idiosyncrasy of the individual functional connectomes is indicated by low Dice overlaps and high MSD from the reference clustering.

The spatial variability existing in the network locations among individuals is reflected in the spreading of the ICN probability maps at the group level. The higher the spreading, the more idiosyncratic are the individuals in a given cohort. Therefore, we further characterized idiosyncrasy using a measure of uncertainty based on the entropy of the group-wise probability maps obtained from clustering. In other words, this descriptor measures how evenly the probability mass is spread among the different ICNs at each location. Entropy is minimized when most of the probability mass is concentrated on a particular network, indicating a location with very low variability among individuals (i.e., the location is assigned to the same ICN in most individuals). On the other hand, entropy is increased when the probability mass at a given location is spread among several ICNs (i.e., the location is assigned to different ICNs across individuals).

### Analysis of idiosyncrasy

Idiosyncrasy was quantified using surface and diffusion distance measures, which we used to perform the following analyses:

- Assessing idiosyncrasy differences between ASD and TD. For the spatial descriptors of idiosyncrasy (i.e., Dice/Jaccard overlap and MSD), general linear models (GLM) predicting each of the idiosyncrasy measures based on group diagnosis were used to assess differences at the network level and cortex-wise. For the latter, overall Dice/Jaccard and MSD were computed as the weighted average of the corresponding scores for each ICN, using the size of the reference networks as weights. All results from our network-level analyses were corrected for multiple comparisons using Benjamini–Hochberg FDR correction^131^. GLMs were also used in the surface-based analysis to study the differences in DD and SDs. For entropy, network-wise differences were analyzed at the group level using two-sample *t*-tests.
- Age effects. To investigate age-specific differences in idiosyncrasy, the surface-based analysis to study differences in DD and SDs was further repeated for children (86 TD and 88 ASD individuals with age <18), and adults (86 TD and 69 ASD individuals with age ≥18) separately.
- Association of idiosyncrasy with ASD symptomatology. Idiosyncrasy descriptors were correlated with ADOS CSS rather than raw ADOS scores since participants with different ages and language abilities undergo assessments using different ADOS modules. For the ABIDE sample used in our work, however, CSS (or the necessary information to derive them) were only available for a small subset of individuals. We therefore resorted to an approximation by using a proxy CSS approach based on social and communication ADOS scores^67^, which are available for all subjects. The proxy CSS were derived by mapping a subject’s age, total ADOS score (social and communication), and ADOS module through a lookup table. Since ABIDE includes modules 2–4, we used the lookup table provided by^65^ for modules 2–3, and the table provided by^66^ for module 4. Results were corrected for multiple comparisons using the FDR procedure. For the correlations of idiosyncrasy with CSS, we *z*-scored the data with respect to TD and regressed out the effects of age, sex, and site prior to performing the correlations.
- Associations to morphology. To assess whether there is an association between these morphological alterations in ASD and functional idiosyncrasy, we correlated group-wise differences in cortical thickness and mean curvature index with surface-based idiosyncrasy measures (i.e., using surface and diffusion distances). We accounted for spatial autocorrelations using nonparametric permutation tests (i.e., spin tests)^51^.

### Gene enrichment analysis

Many risk factors have been associated with neurodevelopmental disorders, with genetic factors playing an important role in the etiology of ASD^132,133^. We, therefore, aimed to investigate the genetic correlates of idiosyncrasy in ASD. Using a similar approach to Neurovault gene decoding tool^57,134^, coherent associations between our idiosyncrasy maps (i.e., t-maps of surface and diffusion distances) and postmortem gene expression patterns from the AIBS were measured to identify the set of genes with significant spatial overlaps. Significant genes were obtained by regressing each gene against our cortical map of idiosyncrasy (e.g., DD) for each donor and using a one-sample *t*-test to determine whether the slopes across all six donors were different from 0. To correct for multiple comparisons, the procedure was repeated by randomly rotating our maps of idiosyncrasy using 1000 spin permutations^51^, which were compared with the original t-statistic to assess gene significance.

Gene expressions for all six donors in the AHBA dataset were obtained using abagen (https://github.com/rmarkello/abagen). Only genes that were consistently expressed across donors (i.e., average inter-donor correlation ≥0.5) were considered for our analyses^58^. Next, we carried out developmental gene expression analysis and disease enrichment analysis. In the former, we identified the genes whose expressions significantly overlapped with our idiosyncrasy maps. The identifiers of this final set of significant genes were then submitted to the cell-type-specific expression analysis (CSEA) developmental expression tool (http://genetics.wustl.edu/jdlab/csea-tool-2/), where they were compared against developmental expression profiles from the BrainSpan dataset (http://www.brainspan.org) to identify the developmental time windows across brain regions in which these genes are expressed. In the second analysis (i.e., disease enrichment), we used a recently published catalog of genes with differential expression information (i.e., fold change values) for autism, schizophrenia, and bipolar disorder^135^. Here, we used robust linear regression to assess the relationship between the t-statistics derived from the previous spatial analysis (i.e., denoting the association of gene expression with our idiosyncrasy map) and their corresponding log fold-changes in each neuropsychiatric disorder^136^. Results for schizophrenia and bipolar disorder were included as baselines, since these disorders share similar genetic variants with ASD^60^. Guanine-cytosine content was used as an additional covariate to control for possible effects related to genome size in microarray data^137,138^.

### Relation to degree centrality

Given the little consensus on the directionality of the connectivity alterations in ASD reported in the literature. Here, our purpose is to investigate the relationship between idiosyncrasy and connectivity alterations to elucidate the role of idiosyncrasy in these connectivity alterations. To study this putative association, we used two different measures of centrality, namely: degree and eigenvector centrality. The first measure is defined as the total number of connections whose linear product-moment correlation coefficients are above a predefined threshold used to eliminate connections with low temporal correlation attributable to signal noise^22,139^. Eigenvector centrality is based on the eigenvector with the largest eigenvalue of the connectivity matrix. Following prior work that used these measures to study connectivity alterations in ASD^22,140^, the threshold for our analyses was set to 0.2.

Since idiosyncrasy is an inherent property that is also present in TD individuals (presumably in a lower degree than in ASD), we first analyzed the relationship of idiosyncrasy with hyper- and hypo-connectivity based on linear product-moment correlations of the statistical t-maps of degree and eigenvector centrality with those of DD and SDs using spin tests^51^. Positive degree centrality values would indicate hyper-connectivity in ASD, whereas negative values indicate hypo-connectivity. The same applies to our idiosyncrasy descriptors, with positive/negative SDs, for instance, pointing out higher/lower deviations from the canonical reference networks relative to TD. Then, we investigated the impact of idiosyncrasy in the potential connectivity alterations when ignoring this phenomenon. Surface-based analysis to find differences in connectivity between ASD and TD was performed based on degree centrality (or eigenvector centrality). This analysis was initially conducted without considering idiosyncrasy and then repeated controlling for idiosyncrasy by incorporating SD and DD as additional covariates to our GLMs.

### Statistics and reproducibility

Groupwise idiosyncrasy differences and correlational analyses controlled for the site, sex, and age effects. For analyses involving spatial idiosyncrasy descriptors (i.e., Dice/Jaccard overlap and SDs), the surface area was further included as a nuisance covariate. For all our surface-based analyses, threshold-free cluster enhancement (TFCE) was used with 10,000 permutations to correct for multiple comparisons across the cortical surfaces^141^. A significance level of 0.05 was used for all statistical tests. Network-level analyses, including associations between idiosyncrasy and CSS, were corrected for multiple comparisons using Benjamini–Hochberg FDR correction^131^. For the correlations of idiosyncrasy with CSS, the data was first *z*-scored with respect to TD and we regressed out the effects of age, sex, and site prior to performing the correlations. Correlation of cortical thickness and mean curvature index with our idiosyncrasy maps was carried out while accounting for spatial autocorrelations using nonparametric permutation tests^51^. For the gene enrichment analysis, significant genes were obtained by regressing each gene against our cortical map of idiosyncrasy for each donor and using a one-sample *t*-test to determine whether the slopes across all six donors were different from 0. We corrected for multiple comparisons by randomly rotating our maps of idiosyncrasy using 1000 spin permutations^5^. The reproducibility of idiosyncrasy differences found using the whole ABIDE data (*n* =  329) was assessed for each acquisition site separately (IP, *n* = 32; NYU, *n* = 126; PITT, *n* = 42; TCD, *n* = 37; USM, *n* = 92) based on surface and diffusion distances. This analysis was also repeated for children (*n* = 174, age <18) and adults (*n* = 155, age ≥18) separately.

### Reporting Summary

Further information on research design is available in the Nature Research Reporting Summary linked to this article.

## Supplementary information

Supplementary Information

Description of Supplementary Files

Supplementary data 1

Reporting Summary

## Data Availability

The imaging and phenotypic data were provided, in part, by the Autism Brain Imaging Data Exchange initiative (ABIDE-I and II; https://fcon_1000.projects.nitrc.org/indi/abide). The specific subsets of data that were used in the present work are available from the authors upon request.

## Code availability

We made all code to run our analyses openly available at http://github.com/MICA-MNI/micaopen.
