# Supplementary material for: Connectivity alterations in autism reflect functional idiosyncrasy
Source: Commun Biol. 2021 Sep 15;4:1078. doi: 10.1038/s42003-021-02572-6 (PMC8443598; doi:10.1038/s42003-021-02572-6)
Supplement: Supplementary file 1 — Supplementary Information [file 42003_2021_2572_MOESM1_ESM.pdf]

CONNECTIVITY ALTERATIONS IN AUTISM REFLECT FUNCTIONAL IDIOSYNCRASY (Supplementary Material)

Oualid Benkarim[1], Casey Paquola[1], Bo-yong Park[1], Seok-Jun Hong[2,3,4], Jessica Royer[1], Reinder Vos de Wael[1], Sara Lariviere[1], Sofie Valk[5,6], Danilo Bzdok[1,7,8], Laurent Mottron[9], Boris Bernhardt[1]

[1]McConnell Brain Imaging Centre, Montreal Neurological Institute and Hospital, McGill University, Montreal, Quebec, Canada; [2]Center for the Developing Brain, Child Mind Institute, New York, NY, USA; [3]Center for Neuroscience Imaging Research, Institute for Basic Science, Sungkyunkwan University, Suwon, South Korea; [4]Department of Biomedical Engineering, Sungkyunkwan University, Suwon, South Korea; [5]Max Planck Institute for Human Cognitive and Brain Sciences, Leipzig, Germany; [6]INM-7, FZ Jülich, Jülich, Germany; [7]Department of Biomedical Engineering, Faculty of Medicine, McGill University, Montreal, Canada; [8]Mila – Quebec Artificial Intelligence Institute, Montreal, Canada; [9]Centre de recherche du CIUSSSNIM et Département de Psychiatrie, Université de Montréal, Montreal, Quebec, Canada

	Scanner	Modality	Sequence	TR (ms)	TE (ms)	TI (ms)	FA	matrix	#Vol	Voxel (mm^3)
IP	Philips	T1w	3D-MPRAGE	2500	5.60		30°	240×240		1.0×1.0×1.0
	Achieva	rs-fMRI	2D-EPI	2700	45.00		90°	63×63	85	3.59×3.65×4.0
NYU	Siemens	T1w	3D-TurboFLASH	2530	3.25	1100	7°	256×256		1.0×1.0×1.3
	Allegra	rs-fMRI	2D-EPI	2000	15.00		90°	80×80	180	3.0×3.0×4.0
PITT	Siemens	T1w	3D-MPRAGE	2100	3.93	1000	7°	269×269		1.1×1.1×1.1
	Allegra	rs-fMRI	2D-EPI	1500	35.00		70°	64×64	200	3.1×3.1×4.0
TCD	Philips	T1w	3D-MPRAGE	3000	3.90	1150	8°	256×256		0.9×0.9×0.9
	Achieva	rs-fMRI	2D-EPI	2000	27.00		90°	80×80	210	3.0×3.0×3.2
USM	Siemens	T1w	3D-MPRAGE	2300	2.91	900	9°	240×256		1.0×1.0×1.2
	TrioTim	rs-fMRI	2D-EPI	2000	28.00		90°	64×64	240	3.4×3.4×3.0

Supplementary Table 1. Scanner and data acquisition settings. Abbreviations: TR, repetition time; TE, echo time; TI, inversion time; FA, flip angle; #Vol, number of volumes; IP, Institut Pasteur/Robert Debré Hospital; NYU, New York University Langone Medical Center; PITT, University of Pittsburgh, School of Medicine; TCD, Trinity Centre for Health Sciences, Trinity College Dublin; USM, University of Utah, School of Medicine.

	N (ASD/TD)	Sex (M/F)	Age	ADOS			
				Comm	Behav	Social	Total
IP	11/21	18/14	20.5±8.8	5.3±2.1	1.6±2.1	9.5±3.5	14.8±5.3
NYU	56/70	121/5	15.0±7.4	3.2±1.7	1.8±1.2	7.6±2.6	10.8±3.9
PITT	20/22	42/-	20.2±7.1	4.2±1.1	2.6±1.2	8.4±2.3	12.7±3.0
TCD	18/19	37/-	15.2±3.3	2.9±0.9	0.2±0.5	5.8±2.4	8.7±2.4
USM	52/40	92/-	22.7±7.7	4.7±1.4	1.8±1.8	8.9±2.5	13.6±3.3
All	157/172	310/19	18.4±8.0	3.9±1.7	1.7±1.5	8.1±2.8	12.0±4.0

Supplementary Table 2. Demographics for each site. First column (N) indicates the number of individuals with autism (ASD) and typically developing (TD); M/F is the number of males/females in each site. Age and Autism Diagnostic Observation Schedule (ADOS) are expressed in mean and standard deviation. Abbreviations: IP, Institut Pasteur/Robert Debré Hospital; NYU, New York University Langone Medical Center; PITT, University of Pittsburgh, School of Medicine; TCD, Trinity Centre for Health Sciences, Trinity College Dublin; USM, University of Utah, School of Medicine. For ADOS: Comm, ADOS communication; Behav, ADOS repeated behavior/interest; and social and total ADOS.

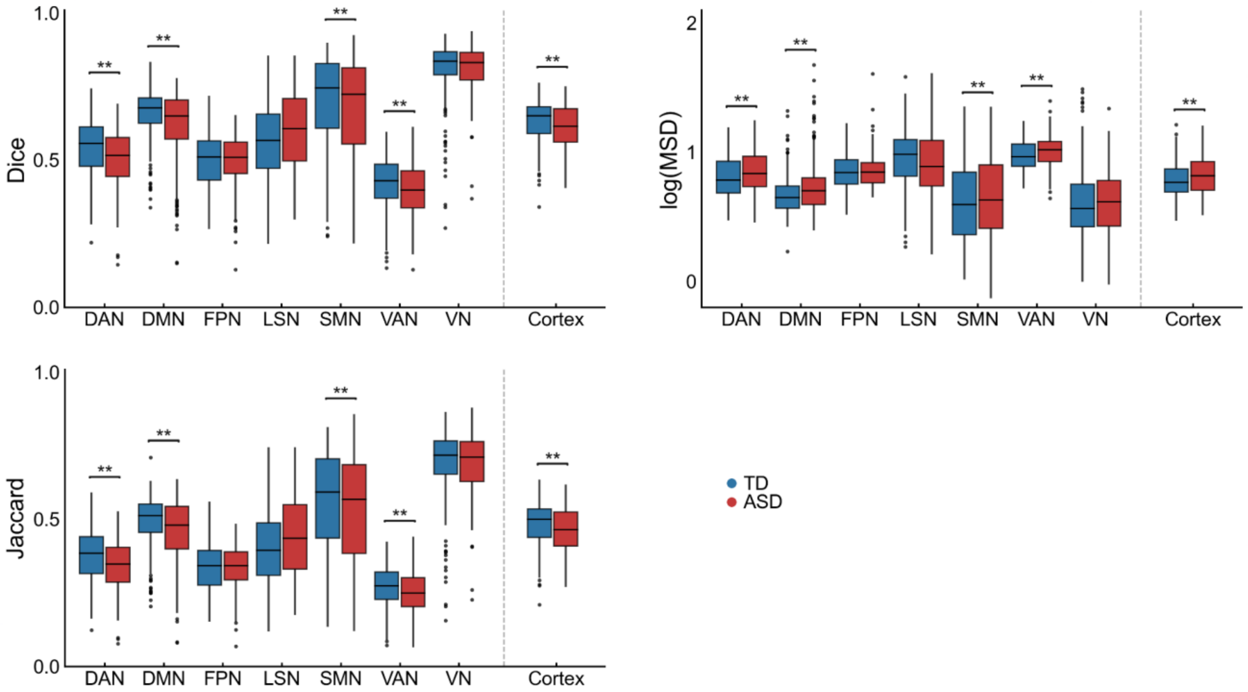

Supplementary Figure 1. Group-wise boxplots of Dice score (top-left), mean surface distance (top-right) and Jaccard index (bottom-left) for each intrinsic connectivity network (i.e., cluster) and across the entire cortex. Statistical significance is indicated with *, ** and ***, respectively denoting p<0.05, p<0.01, and p<0.001 after FDR correction across 7 networks. Abbreviations: DAN, dorsal attention network; DMN, default mode network; FPN, frontoparietal network; LSN, limbic system network; SMN, somatomotor network; VAN, ventral attention network; VN, visual network. Boxes denote the interquartile (IQR) between the first and third quartiles, and the line inside denotes the median. Whiskers extend to points that lie within 1.5 IQRs of the lower and upper quartiles.

	**ICN size**
DAN	t=-0.20, p=0.840
DMN	t=-2.06, p=0.093
FPN	t=2.44, p=0.053
LSN	t=2.62, p=0.053
SMN	t=-1.72, p=0.151
VAN	t=0.64, p=0.725
VN	t=0.49, p=0.725

Supplementary Table 3. Intrinsic connectivity network (ICN) size differences between ASD and TD after controlling for age, sex and site. No statistically significant differences were found.

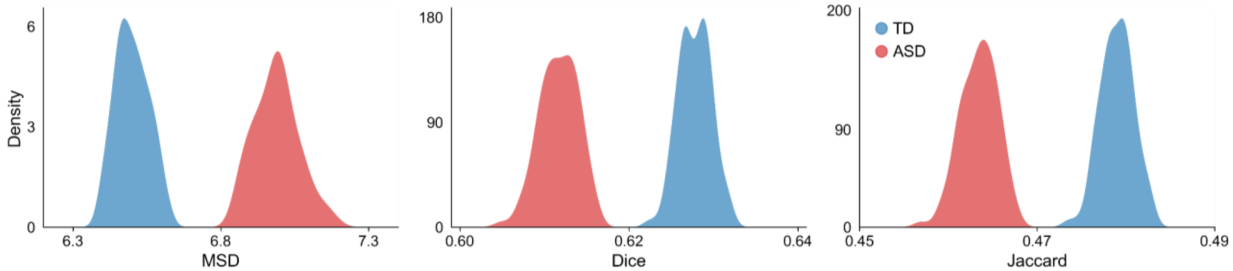

Supplementary Figure 2. Distributions of average cortex-wide mean surface distance (MSD), Dice and Jaccard overlap scores based on reference embeddings built from different subsets of subjects chosen at random.

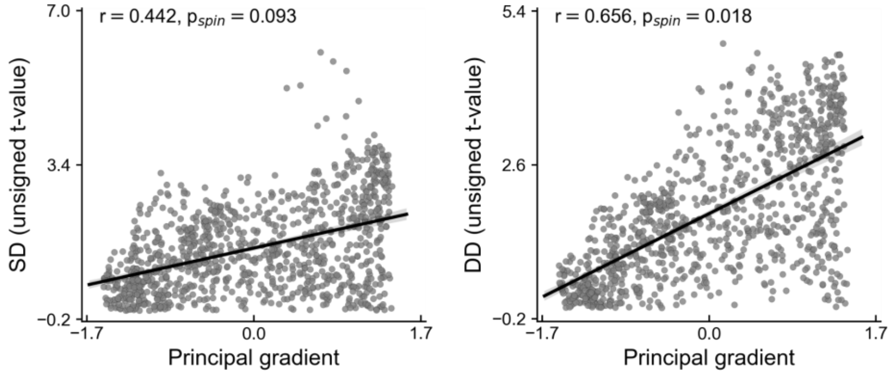

Supplementary Figure 3. Emergence of idiosyncrasy along the human cortical hierarchical organization. From left to right, association of idiosyncrasy maps in terms of unsigned t-maps of surface distance (SD) and diffusion distance (DD) with the principal connectivity gradient, where negative values represent sensory/motor regions and positive values correspond to transmodal cortices. Shaded areas around the regression lines denote 95% confidence interval.

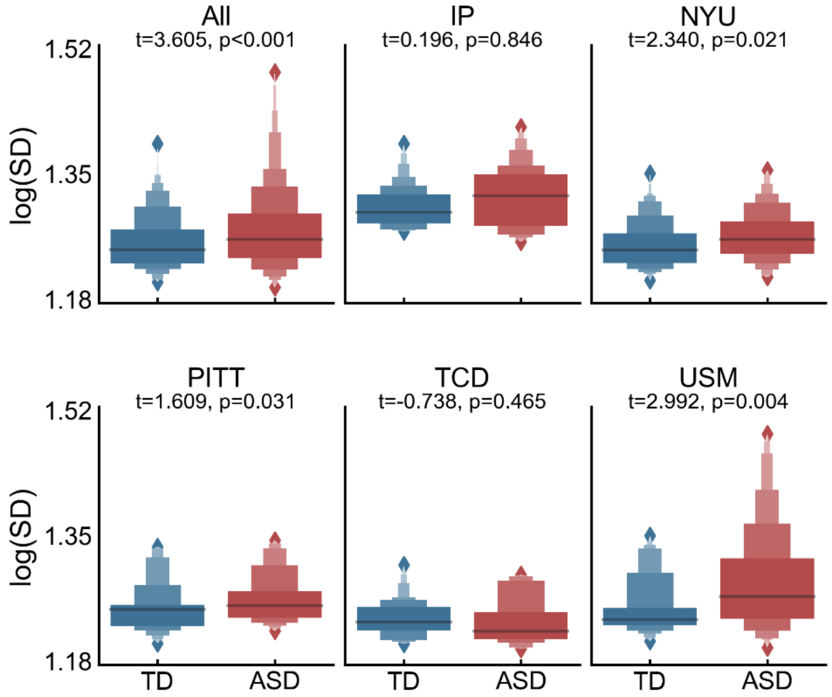

Supplementary Figure 4. Site-specific idiosyncrasy differences. Distributions of mean surface distance in TD and ASD for all included sites in the ABIDE I and II datasets and for each site individually.

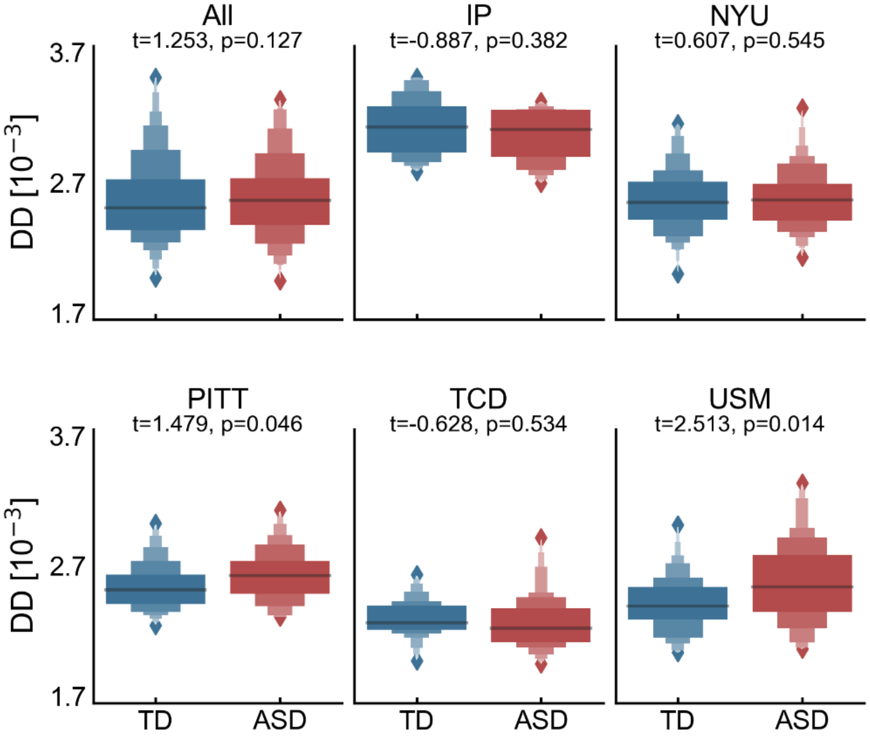

Supplementary Figure 5. Site-specific idiosyncrasy differences. Distributions of mean diffusion distance in TD and ASD for all included sites in the ABIDE I and II datasets and for each site individually.

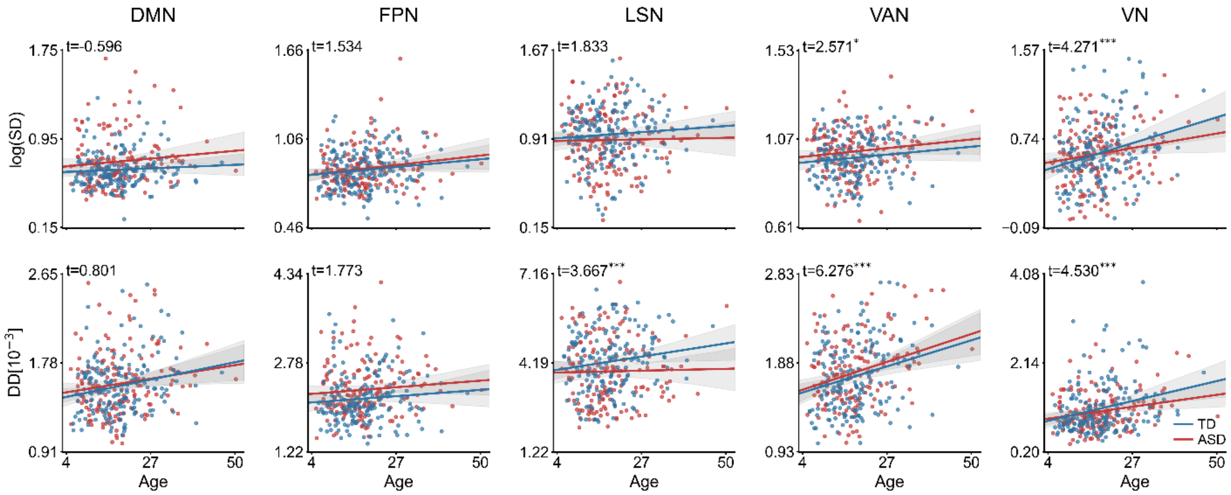

Supplementary Figure 6. Relationship of spatial shifting with age. Average surface (top) and diffusion (bottom) distances in ASD and TD versus age for default mode (DMN), frontoparietal (FPN), limbic system (LSN), ventral attention (VAN) and visual (VN) networks. Remaining networks are available in the main document. Statistical significance is indicated with *, ** and ***, respectively denoting p<0.05, p<0.01, and p<0.001 after FDR correction across 7 networks. Shaded areas around the regression lines denote 95% confidence interval.

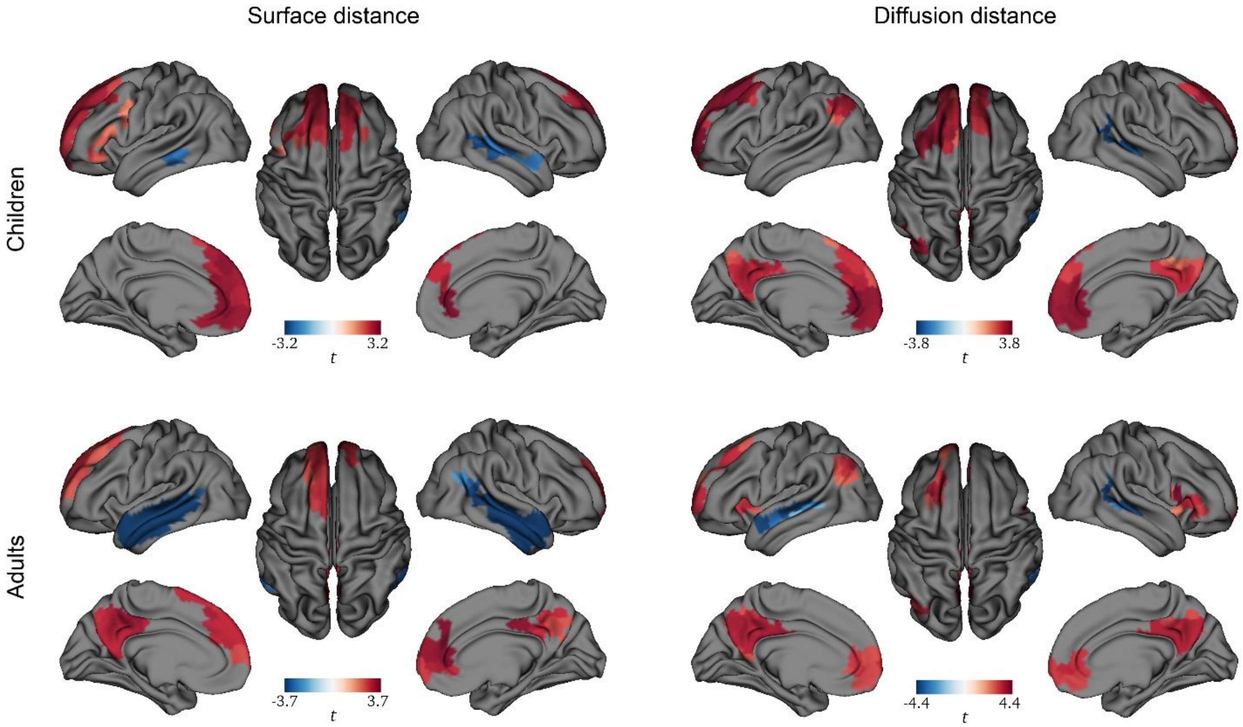

Supplementary Figure 7. Idiosyncrasy differences, using surface (left) and diffusion (right) distances, between ASD and TD in children (i.e., <18 years) and adults (≥18 years). Significance maps obtained using permutation-based thresholding with 10000 permutations.

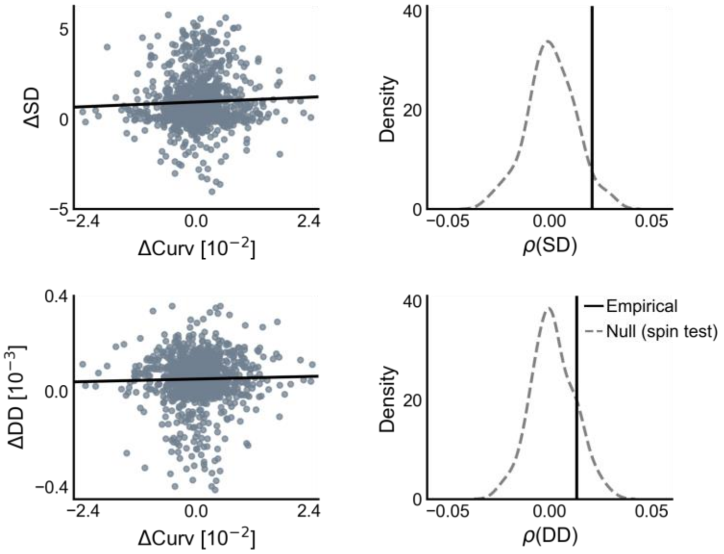

Supplementary Figure 8. Correlation of group-wise differences in surface (top) and diffusion (bottom) distances with Mean Curvature Index (Curv), and comparison of empirical correlation with the null distribution obtained using 1000 spin permutation tests to account for spatial autocorrelation.

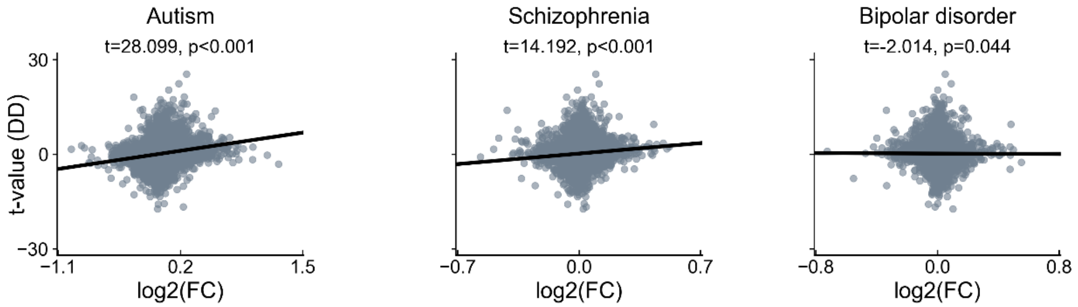

Supplementary Figure 9. Associations of gene expression in neuropsychiatric disorders, where log2(FC) stands for log2 fold-change of the genes in each disorder and the vertical axis indicates the significance of the relationship strength of the genes with idiosyncrasy in terms of diffusion distance (DD).

Gene	Name
AGO1	argonaute RISC component 1
ALDH1A3	aldehyde dehydrogenase 1 family member A3
ANK1	ankyrin 1
ANKH	ANKH inorganic pyrophosphate transport regulator
BEND6	BEN domain containing 6
CCNO	cyclin O
COL5A1	collagen type V alpha 1 chain
CPLX1	complexin 1
DMKN	Dermokine
ECM1	extracellular matrix protein 1
ESRRG	estrogen related receptor gamma
GLCCI1	glucocorticoid induced 1
GPCPD1	glycerophosphocholine phosphodiesterase 1
HS3ST1	heparan sulfate-glucosamine 3-sulfotransferase 1
KCNAB3	potassium voltage-gated channel subfamily A regulatory beta subunit 3
KCNC1	potassium voltage-gated channel subfamily C member 1
KCNC3	potassium voltage-gated channel subfamily C member 3
LINC00599	MIR124-1 host gene
LYSMD4	LysM domain containing 4
MAGI2-AS3	MAGI2 antisense RNA 3
MET	MET proto-oncogene, receptor tyrosine kinase
MIR124-2HG	MIR124-2 host gene
MIR29B2CHG	MIR29B2 and MIR29C host gene
NFIC	nuclear factor I C
NIPAL2	NIPA like domain containing 2
NOXO1	NADPH oxidase organizer 1
OIP5-AS1	OIP5 antisense RNA 1
RAD54B	RAD54 homolog B
SCN1B	sodium voltage-gated channel beta subunit 1
SCRT1	scratch family transcriptional repressor 1
SEMA7A	semaphorin 7A (John Milton Hagen blood group)
SHD	Src homology 2 domain containing transforming protein D
SLC25A37	solute carrier family 25 member 37
UPP1	uridine phosphorylase 1
WDR97	WD repeat domain 97

Supplementary Table 4. List of genes (symbol and name) significantly associated with idiosyncrasy maps of surface and diffusion distances.

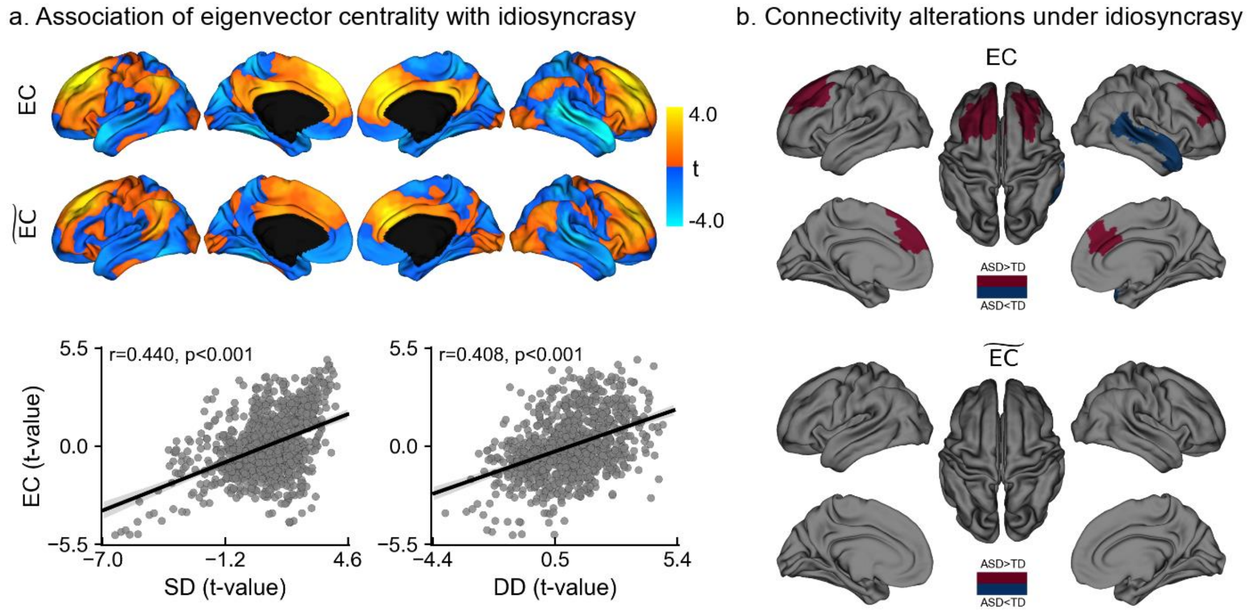

Supplementary Figure 10. Association of eigenvector centrality with idiosyncrasy. **a**) Statistical t-maps (top) of areas showing differences in eigenvector centrality between ASD and TD before (i.e., EC) and after controlling for idiosyncrasy (i.e., $\widetilde{EC}$), and Pearson's correlation (bottom) of EC t-map with t-maps of surface (SD) and diffusion distances (DD). **b**) Regions showing significant EC increases (red) and decreases (blue) in ASD before (top) and after (bottom) controlling for idiosyncrasy. Idiosyncrasy is represented with SD and DD as additional covariates. Shaded areas around the regression lines denote 95% confidence interval. No significant differences were found after controlling for idiosyncrasy.

	SD	DD
DAN	$t = 1.99^*$	$t = 3.98^*$
DMN	$t = 3.34^*$	$t = 5.39^*$
FPN	$t = 1.95$	$t = 5.84^*$
LSN	$t = 1.51$	$t = 4.90^*$
SMN	$t = -2.16^*$	$t = 4.80^*$
VAN	$t = 1.71^*$	$t = 3.45^*$
VN	$t = 2.09^*$	$t = 5.75^*$

Supplementary Table 5. Relationship of idiosyncrasy, in terms of network-wise surface (SD) and diffusion (DD) distances, with average eigenvector centrality for each intrinsic connectivity network. Significant associations after FDR correction are denoted with *.
