# Supplementary material for: Connectivity alterations in autism reflect functional idiosyncrasy
Source: Commun Biol. 2021 Sep 15;4:1078. doi: 10.1038/s42003-021-02572-6 (PMC8443598; doi:10.1038/s42003-021-02572-6)
Supplement: Supplementary file 2 — Description of Supplementary Files [file 42003_2021_2572_MOESM2_ESM.pdf]

Description of Additional Supplementary Files

File name: Supplementary Data 1
Description: Description: Source data for graphs and charts.
