# Supplementary material for: Connectivity alterations in autism reflect functional idiosyncrasy
Source: Commun Biol. 2021 Sep 15;4:1078. doi: 10.1038/s42003-021-02572-6 (PMC8443598; doi:10.1038/s42003-021-02572-6)
Supplement: Supplementary file 4 — Reporting Summary [file 42003_2021_2572_MOESM4_ESM.pdf]

nature portfolio

Corresponding author(s): Boris Bernhardt

Last updated by author(s): Aug 3, 2021

Reporting Summary

Nature Portfolio wishes to improve the reproducibility of the work that we publish. This form provides structure for consistency and transparency in reporting. For further information on Nature Portfolio policies, see our Editorial Policies and the Editorial Policy Checklist.

Statistics

For all statistical analyses, confirm that the following items are present in the figure legend, table legend, main text, or Methods section.

n/a	Confirmed	
☐	☒	The exact sample size (*n*) for each experimental group/condition, given as a discrete number and unit of measurement
☐	☒	A statement on whether measurements were taken from distinct samples or whether the same sample was measured repeatedly
☐	☒	The statistical test(s) used AND whether they are one- or two-sided<br>*Only common tests should be described solely by name; describe more complex techniques in the Methods section.*
☐	☒	A description of all covariates tested
☐	☒	A description of any assumptions or corrections, such as tests of normality and adjustment for multiple comparisons
☐	☒	A full description of the statistical parameters including central tendency (e.g. means) or other basic estimates (e.g. regression coefficient) AND variation (e.g. standard deviation) or associated estimates of uncertainty (e.g. confidence intervals)
☐	☒	For null hypothesis testing, the test statistic (e.g. *F*, *t*, *r*) with confidence intervals, effect sizes, degrees of freedom and *P* value noted<br>*Give P values as exact values whenever suitable.*
☒	☐	For Bayesian analysis, information on the choice of priors and Markov chain Monte Carlo settings
☒	☐	For hierarchical and complex designs, identification of the appropriate level for tests and full reporting of outcomes
☐	☒	Estimates of effect sizes (e.g. Cohen's *d*, Pearson's *r*), indicating how they were calculated

Our web collection on statistics for biologists contains articles on many of the points above.

Software and code

Policy information about availability of computer code

Data collection	We studied imaging and phenotypic data of 157 individuals with autism and 172 typically developing controls from the Autism Brain Imaging Data Exchange initiative (ABIDE I and II; http:/fcon_1000.projects.nitrc.org/indi/abide).
Data analysis	T1-weighted data preprocessing using FreeSurfer (v5.1; http://surfer.nmr. mgh.harvard.edu/)<br>Resting-state fMRI preprocessing using C-PAC (https://fcp-indi.github.io)<br>Connectome manifold generation using BrainSpace (https://github.com/MICA-MNI/BrainSpace).<br>Gene enrichment analysis was performed with Cell-type Specific Expression Analysis (CSEA) developmental expression tool (http://genetics.wustl.edu/jdlab/csea-tool-2/), and abagen (https://github.com/rmarkello/abagen).

For manuscripts utilizing custom algorithms or software that are central to the research but not yet described in published literature, software must be made available to editors and reviewers. We strongly encourage code deposition in a community repository (e.g. GitHub). See the Nature Portfolio guidelines for submitting code & software for further information.

Data

Policy information about availability of data

All manuscripts must include a data availability statement. This statement should provide the following information, where applicable:

- Accession codes, unique identifiers, or web links for publicly available datasets
- A description of any restrictions on data availability
- For clinical datasets or third party data, please ensure that the statement adheres to our policy

The imaging and phenotypic data were provided, in part, by the Autism Brain Imaging Data Exchange initiative (ABIDE-I and II; https://fcon_1000.projects.nitrc.org/indi/abide). The specific subsets of data that were used in the present work are available from the authors upon request.

Field-specific reporting

Please select the one below that is the best fit for your research. If you are not sure, read the appropriate sections before making your selection.

[x] Life sciences [ ] Behavioural & social sciences [ ] Ecological, evolutionary & environmental sciences

For a reference copy of the document with all sections, see nature.com/documents/nr-reporting-summary-flat.pdf

Life sciences study design

All studies must disclose on these points even when the disclosure is negative.

Sample size	We studied rs-fMRI data from both waves of the openly-shared Autism Brain Imaging Data Exchange initiative (ABIDE I and II; http://fcon_1000.projects.nitrc.org/indi/abide). Our dataset was composed of 329 subjects (157/172 ASD/TD, with mean±SD age in years = 18.4 ±8.2/18.4±7.7) from 5 different sites: (1) NYU Langone Medical Center (NYU, 35/51 ASD/TD from ABIDE-I, and 21/19 from ABIDE-II); (2) University of Utah, School of Medicine (USM, 49/37 ASD/TD); (3) University of Pittsburgh, School of Medicine (PITT, 19/20 ASD/TD); (4) Trinity Centre for Health Sciences, Trinity College Dublin (TCD, 12/16 ASD/TD); and (5) Institut Pasteur/Robert Debré Hospital (IP, 11/21 ASD/TD).
Data exclusions	Individuals with ASD were diagnosed by an in-person interview with clinical experts and gold standard diagnostics of the Autism Diagnostic Observation Schedule, ADOS (Lord et al., 2000) and/or Autism Diagnostic Interview-Revised (ADI-R) (Lord et al., 1994). TD individuals did not have any history of mental disorders. For all groups, participants who had genetic disorders associated with autism (i.e., Fragile X), contraindications to MRI scanning, and pregnancy were excluded. For our study, we selected those sites with ≥10 individuals per group and with both children and adults. After detailed quality control, only cases with acceptable T1-weighted (T1w) MRI, surface-extraction, and head motion in rs-fMRI were included in our analyses.
Replication	N/A
Randomization	Our sample consisted of 157 individuals with autism and 172 neurotypical controls obtained from the 5 independent sites from the Autism Brain Imaging Data Exchange initiative (ABIDE-II; http:/fcon_1000.projects.nitrc.org/indi/abide). We therefore controlled for site in all our analyses.
Blinding	Individuals with autism were diagnosed by an in-person interview with clinical experts and gold standard diagnostics of Autism Diagnostic Observation Schedule (ADOS) and/or Autism Diagnostic Interview-Revised (ADI-R). Investigators for data analysis were blinded during data collection.

Reporting for specific materials, systems and methods

We require information from authors about some types of materials, experimental systems and methods used in many studies. Here, indicate whether each material, system or method listed is relevant to your study. If you are not sure if a list item applies to your research, read the appropriate section before selecting a response.

Materials & experimental systems

n/a	Involved in the study	
[x]	[ ]	Antibodies
[x]	[ ]	Eukaryotic cell lines
[x]	[ ]	Palaeontology and archaeology
[x]	[ ]	Animals and other organisms
[ ]	[x]	Human research participants
[x]	[ ]	Clinical data
[x]	[ ]	Dual use research of concern

Methods

n/a	Involved in the study	
[x]	[ ]	ChIP-seq
[x]	[ ]	Flow cytometry
[ ]	[x]	MRI-based neuroimaging

Human research participants

Policy information about studies involving human research participants

Population characteristics	- Number (Autism/Control): 11/21 for IP site, 56/70 NYU, 20/22 PITT, 18/19 TCD, 52/40 USM<br>- Age: 20.5 (SD 8.8) for IP site, 15.0 (SD 7.4) NYU, 20.2 (SD 7.1) PITT, 15.2 (SD 3.3) TCD, 22.7 (SD 7.7) USM<br>- Sex (male:female): 18:14 for IP site, 121:5 NYU, 42:- PITT, 37:- TCD, 92:- USM<br>- ADOS Total: 14.8 (SD 5.3) for IP site, 10.8 (SD 3.9) NYU, 12.7 (SD 3.0) PITT, 8.7 (SD 2.4) TCD, 13.6 (SD 3.3) USM
Recruitment	Our sample consisted of 157 individuals with autism and 172 neurotypical controls obtained from the 5 independent sites of (1) NYU Langone Medical Center (NYU); (2) University of Utah, School of Medicine (USM); (3) University of Pittsburgh, School of Medicine (PITT); (4) Trinity Centre for Health Sciences, Trinity College Dublin (TCD); and (5) Institut Pasteur/Robert Debré Hospital (IP) from the Autism Brain Imaging Data Exchange initiative (ABIDE-II; http:/fcon_1000.projects.nitrc.org/indi/abide).
Ethics oversight	The ABIDE data collections were performed in accordance with local Institutional Review Board guidelines. In accordance with HIPAA guidelines and 1000 Functional Connectomes Project/INDI protocols, all ABIDE datasets have been fully anonymized, with no protected health information included.

Note that full information on the approval of the study protocol must also be provided in the manuscript.

Magnetic resonance imaging

Experimental design

Design type	Resting-state
Design specifications	Resting-state
Behavioral performance measures	Resting-state

Acquisition

Imaging type(s)	T1-weighted, resting-state fMRI
Field strength	3T
Sequence & imaging parameters	IP site<br>T1-weighted: 3D-MPRAGE (TR = 2500 ms; TE = 5.60 ms; flip angle = 30°; matrix = 240×240; and voxel size = 1.0×1.0×1.0 mm3)<br>rs-fMRI: 2D EPI (TR = 2700 ms; TE = 45.00 ms; flip angle = 90°; matrix = 63×63; number of volumes = 85; and voxel size = 3.59×3.65×4.0 mm3)<br><br>NYU site<br>T1-weighted: 3D-MPRAGE (TR = 2530 ms; TE = 3.25 ms; TI = 1100 ms; flip angle = 7°; matrix = 256×256; and voxel size = 1.0×1.0×1.3 mm3)<br>rs-fMRI: 2D EPI (TR = 2000 ms; TE = 15.00 ms; flip angle = 90°; matrix = 80×80; number of volumes = 180; and voxel size = 3.0×3.0×4.0 mm3)<br><br>PITT site<br>T1-weighted: 3D-MPRAGE (TR = 2100 ms; TE = 3.93 ms; TI = 1000 ms; flip angle = 7°; matrix = 269×269; and voxel size = 1.1×1.1×1.1 mm3)<br>rs-fMRI: 2D EPI (TR = 1500 ms; TE = 35.00 ms; flip angle = 70°; matrix = 64×64; number of volumes = 200; and voxel size = 3.1×3.1×4.0 mm3)<br><br>TCD site<br>T1-weighted: 3D-MPRAGE (TR = 3000 ms; TE = 3.90 ms; TI = 1150 ms; flip angle = 8°; matrix = 256×256; and voxel size = 0.9×0.9×0.9 mm3)<br>rs-fMRI: 2D EPI (TR = 2000 ms; TE = 27.00 ms; flip angle = 90°; matrix = 80×80; number of volumes = 210; and voxel size = 3.0×3.0×3.2 mm3)<br><br>USM site<br>T1-weighted: 3D-MPRAGE (TR = 2300 ms; TE = 2.91 ms; TI = 900 ms; flip angle = 9°; matrix = 240×256; and voxel size = 1.0×1.0×1.2 mm3)<br>rs-fMRI: 2D EPI (TR = 2000 ms; TE = 28.00 ms; flip angle = 90°; matrix = 64×64; number of volumes = 240; and voxel size = 3.4×3.4×3.0 mm3)
Area of acquisition	whole brain

Diffusion MRI ☐ Used ☒ Not used

Preprocessing

Preprocessing software	T1-weighted: FreeSurfer (gradient nonuniformity correction, skull stripping, intensity normalization, tissue segmentation, white and pial surfaces generation, topology correction, inflation, and spherical registration to fsaverage)<br>Rs-fMRI: C-PAC (slice timing and head motion correction, skull stripping, intensity normalization, nuisance variables removal, linear/quadratic trends removal, band-pass filtering, co-registeration to T1-weighted data in MNI standard space, map to subject-specific midthickness surfaces and resample to the Conte69 template, and surface-based spatial smoothing)
Normalization	rs-fMRI data were coregistered to T1-weighted data in MNI152 space with boundary-based rigid-body and non-linear transformations. The rs-fMRI data we mapped to subject-specific midthickness surfaces and resampled to the Conte69 template.
Normalization template	MNI template in volume space and Conte69 template in surface space
Noise and artifact removal	T1-weighted: Gradient nonuniformity correction.<br>rs-fMRI: Nuiscance variables of head motion, average white matter and cerebrospinal fluid signal, and linear/quadratic trends were removed using CompCor. Band-pass filtering between 0.01 and 0.1 Hz was applied.
Volume censoring	Data with a framewise displacement of rs-fMRI data > 0.3 mm were excluded

Statistical modeling & inference

Model type and settings	Multivariate analyses controlled for age, sex, and site in addition to including the group factor.
Effect(s) tested	DR corrected p-values

Specify type of analysis: ☐ Whole brain ☒ ROI-based ☐ Both

Anatomical location(s): Schaefer parcellation

Statistic type for inference (See Eklund et al. 2016)	ROI-wise
Correction	permutation tests

Models & analysis

n/a	Involved in the study
☐	☒ Functional and/or effective connectivity
☒	☐ Graph analysis
☐	☒ Multivariate modeling or predictive analysis

Functional and/or effective connectivity	Pearson's correlation
Multivariate modeling and predictive analysis	After controlling for age, sex, and site, multivariate analyses compared idiosyncrasy descriptors of individuals with autism and neurotypicals. Idiosyncrasy descriptors we derived from rs-fMRI data based on diffusion maps and GMM clustering. We used two descriptors of idiosyncrasy: 1) surface distance (i.e., SD), which was computed for each point as the geodesic distance to the closest point in the corresponding reference network, and 2) diffusion distance, which is approximated using the Euclidean distance in the eigenspace between points of each individual to the reference embedding.
